# A spontaneously evolving multifunctional interphase enables durable cycling in solid-state lithium metal batteries

**DOI:** 10.1039/d6sc04429h

**Published:** 2026-08-05

**Authors:** Haijie Lin, Xiang Xie, Fenghua Zheng, Wei Liu, Xinyou He, Zhiming Xiao, Wei Xu, Fenghua Ding, Xinghui Liang, Baishan Chen, Lei Ming, Zhang Lin, Xing Ou

**Affiliations:** a Engineering Research Center of the Ministry of Education for Advanced Battery Materials, School of Metallurgy and Environment, Central South University Changsha 410083 P.R. China liangxh@csu.edu.cn minglei666@csu.edu.cn ouxing@csu.edu.cn; b Guangxi Key Laboratory of Low Carbon Energy Materials, School of Chemistry and Pharmaceutical Sciences, Guangxi Normal University Guilin 541004 PR China; c Quzhou Huayou Cobalt New Mat. Co. Ltd Quzhou 324000 P.R. China; d Institute for Advanced Study, Central South University Changsha 410083 China 221333@csu.edu.cn

## Abstract

NASICON-type Li_1.3_Al_0.3_Ti_1.7_(PO_4_)_3_ (LATP) is a highly attractive solid electrolyte for solid-state lithium batteries, yet direct contact with Li metal readily induces Ti^4+^ reduction, electron leakage, heterogeneous interphase growth, and stress concentration, which collectively trigger interfacial degradation and dendrite penetration. Herein, a PVDF coating loaded with modified AlN is introduced at the LATP|Li interface to address the intrinsic interfacial instability of LATP|Li. Through PVDF defluorination, the coating forms an F–N bifunctional inorganic filler framework anchored within the PVDF matrix. Upon *in situ* reaction with Li metal, this framework yields an F–N bifunctional mosaic-structured interphase composed of coordinated LiF/Li_*x*_Al and Li_3_N/Li_*x*_Al heterogeneous microdomains, synergistically blocking electron leakage, facilitating Li^+^ transport, homogenizing interfacial charge distribution, regulating Li deposition, and accommodating mechanical stress. Consequently, the modified interface delivers durable cycling performance in LFP full cells, achieving 141.8 mA h g^−1^ after 800 cycles with 96.62% capacity retention at 0.5C. Notably, the evolved interphase integrates electrochemical and chemo-mechanical functions to enable stable cycling of NCM811 full cells, delivering an initial discharge capacity of 186.5 mA h g^−1^ at 0.5C with 82.14% capacity retention after 150 cycles. This work provides a design strategy for constructing durable cooperative interphases for solid-state batteries.

## Abstract 2

Surface-modified AlN first induces PVDF defluorination to create an F–N precursor, which then evolves with Li metal into a LiF/Li_*x*_Al–Li_3_N/Li_*x*_Al mosaic interphase for durable solid-state Li-metal batteries.

## Graphical abstract

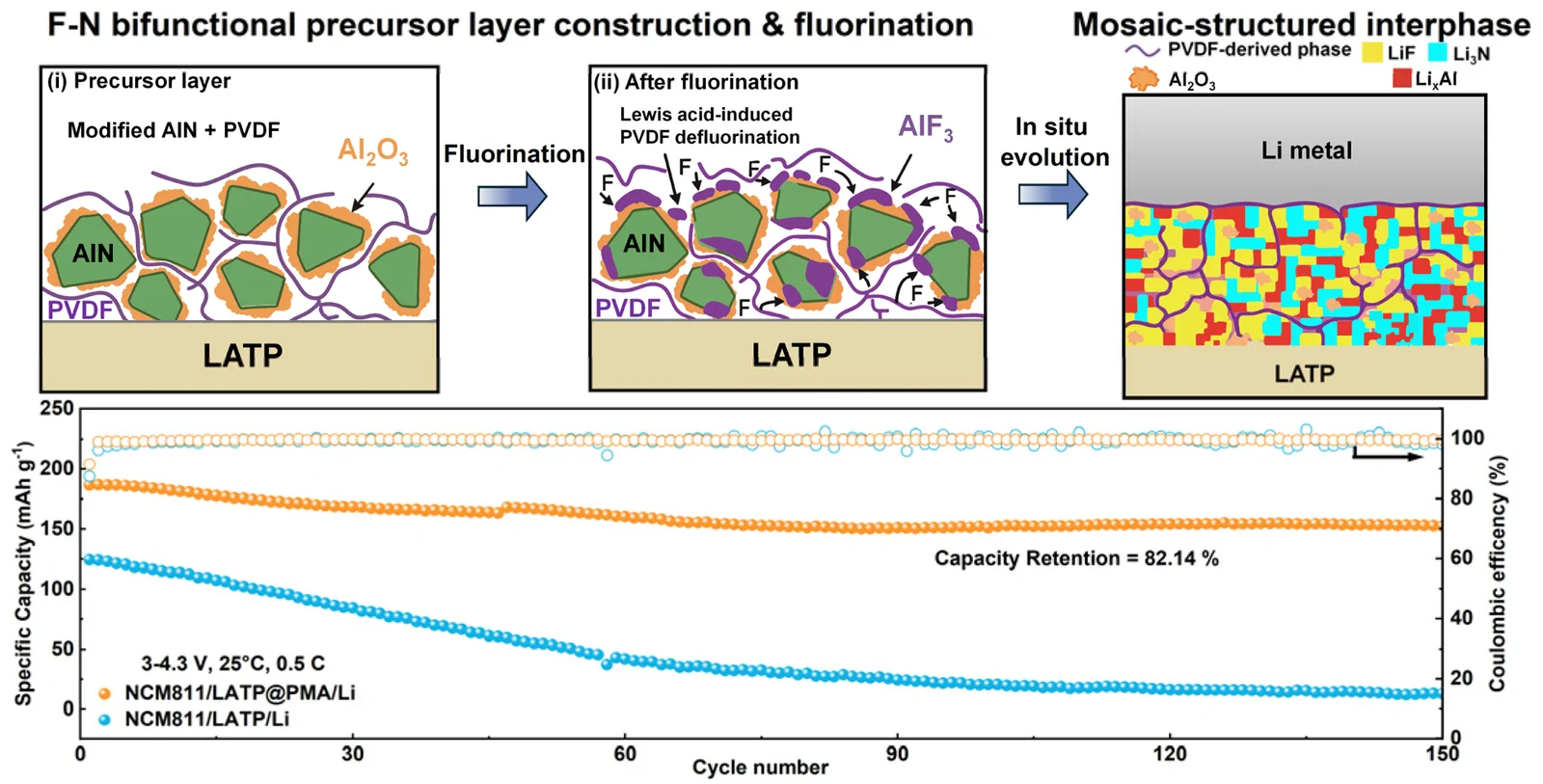

## Introduction

All-solid-state lithium batteries (ASSLBs) have emerged as promising energy-storage systems because of their intrinsic safety and potential for high energy density.^1,2^ Among the inorganic solid electrolytes investigated so far, NASICON-type Li_1.3_Al_0.3_Ti_1.7_(PO_4_)_3_ (LATP) is particularly attractive because of its relatively high room-temperature Li^+^ conductivity, wide electrochemical window, favorable air stability, and low-cost processability.^3,4^ However, direct contact with Li metal remains highly problematic because the reducible Ti^4+^ species in LATP can be converted into lower-valence states, giving rise to a reactive mixed ionic/electronic conducting interphase that facilitates electron leakage and sustained side reactions.^5–7^ Meanwhile, such interfacial reactions are usually spatially heterogeneous, and the accompanying interphase growth and local volume change can generate pronounced stress concentration between reacted and unreacted regions, thereby inducing crack formation and structural deterioration of the electrolyte.^8,9^ The resulting unstable and heterogeneous interphase further promotes localized Li deposition and eventual dendritic penetration.^10–12^

Recent studies on NASICON-type solid electrolytes have increasingly relied on interfacial layers to stabilize the reactive Li/electrolyte interface, with their effectiveness mainly demonstrated in LiFePO_4_ (LFP)-based full cells.^13,14^ For example, a thin BN-based coating has been introduced on LATP to block direct LATP|Li contact while maintaining Li^+^ transport and lowering interfacial resistance through *in situ* Li–N formation.^15^ An ultrathin single-ion-conducting polymer layer has also been employed to guide homogeneous Li^+^ flux and improve LATP|Li interfacial contact,^16^ whereas an ultrathin Al buffer layer has been shown to enhance interfacial lithiophilicity and homogenize charge distribution.^17^ These representative studies highlight that stabilizing NASICON|Li interfaces relies on multiple key requirements, including sustaining Li^+^ transport, blocking electron leakage, and regulating Li deposition. Yet these functions have mostly been realized separately, rather than being integrated into a single interfacial design. More importantly, as lithium batteries continue to pursue higher energy density, cathode development is increasingly shifting toward Ni-rich layered materials such as LiNi_0.8_Co_0.1_Mn_0.1_O_2_ (NCM811).^18–20^ In such systems, the key challenge arises from the more pronounced volume fluctuation associated with anisotropic lattice-parameter evolution during repeated lithiation and delithiation, which can more readily reduce cathode/electrolyte contact and disturb ionic transport pathways.^21,22^ Under rigid solid-state constraints, where mechanical perturbation is difficult to relax, these effects may further induce crack formation and structural deterioration of the brittle solid electrolyte.^9,23^ Therefore, for stable-cycling, high-energy-density LATP-based batteries, interfacial layers should additionally provide stress-buffering capability to tolerate such fluctuation while preserving stable interfacial contact.

To meet these coupled requirements, extensive efforts have been devoted to interfacial engineering in ASSLBs, where the construction of multifunctional interlayers generally relies on deliberately designed interfacial chemistry and structural organization.^24–26^ In LATP-based systems, recent studies have likewise begun to pursue multifunctional interfacial design, where coupled interfacial functions typically need to be achieved through specifically designed interfacial chemistry and conversion pathways. For example, a polymer-in-salt artificial protection layer has been reported to sustain Li^+^ transport, block electron leakage, and regulate Li deposition;^27^ a Kevlar aramid nanofiber membrane combined with an *in situ* polymerized solidified electrolyte has been used to construct a coating that sustains Li^+^ transport, blocks electron leakage, and maintains stable interfacial contact during cycling;^28^ and an SnCl_4_ precursor has been employed to generate a LiCl/Li_*x*_Sn interlayer *via in situ* electrochemical conversion, showing that Li^+^ transport, electron blocking, and Li-deposition regulation can be integrated through reaction-derived interfacial chemistry.^29^ Multifunctional interfacial design has been explored more extensively in other solid-electrolyte systems, where more complete interfacial functional coupling has been achieved, but typically through substantially more elaborate interfacial construction. Representative examples include the salt-based organic–inorganic nanocomposite interphase generated through *in situ* electrolyte decomposition on Li metal,^30^ the Li–Al–Cl stratified interface constructed through a sublimating-winding-rolling process,^31^ and the flexible electron-blocking shield produced through an *in situ* substitution reaction between a polymer coating and molten Li.^32^ These studies collectively indicate that the major challenge lies in constructing a cooperative interphase in which multiple interfacial functions are integrated to achieve coupled mechanical and chemical stabilization. Therefore, for LATP-based systems, what remains particularly desirable is a simple and direct route that can enable a readily introduced coating to evolve into such a multifunctional cooperative interphase, simultaneously enabling Li^+^ transport, electron blocking, homogeneous Li deposition, and stress accommodation.

Herein, we report a simple coating-enabled interfacial strategy in which a modified AlN-loaded PVDF coating is introduced at the LATP|Li interface to address the intrinsic interfacial instability of LATP|Li. During coating formation, modified AlN induces PVDF defluorination and builds an F–N bifunctional inorganic filler framework within the PVDF matrix, which subsequently undergoes *in situ* evolution upon contact with Li metal to form a mosaic-structured interphase composed of coordinated LiF/Li_*x*_Al and Li_3_N/Li_*x*_Al heterogeneous microdomains. This evolved interphase enables electron blocking, favorable Li^+^ transport, homogenized interfacial charge distribution, and regulated Li deposition, while also improving stress accommodation and contact stability during cycling. As a result, LFP full cells with the modified interface deliver 141.8 mA h g^−1^ after 800 cycles with 96.62% capacity retention at 0.5C. Notably, NCM811 full cells with the same interfacial design remain effective under more demanding conditions, maintaining 82.14% capacity retention after 150 cycles at 0.5C. This study demonstrates a design strategy for high-performance LATP-based solid-state batteries that leverages spontaneous interface evolution to create a persistent, synergistic effect at the solid electrolyte|Li metal anode.

## Results and discussion

To realize the spontaneous construction of a cooperative LATP|Li interphase, a modified AlN/PVDF precursor coating was designed on LATP in this work (Fig. 1). For simplicity, the modified AlN obtained by annealing pristine AlN at 950 °C for 6 h was denoted as MA, and the corresponding PVDF/AlN and PVDF/MA coatings were denoted as PA and PMA, respectively. In this design, PVDF served as the fluorine source and contributed to the compliant character of the interphase, while MA provided chemically active sites that induced and utilized PVDF defluorination. Through this coupling, fluorine was transferred from the polymer environment to the inorganic component, leading to the formation of an F–N bifunctional precursor coating with strengthened interfacial coupling on LATP. Furthermore, during electrochemical cycling, this precursor layer underwent *in situ* conversion into an interphase enriched with co-distributed LiF, Li_3_N, and Li_*x*_Al related species, thereby establishing heterogeneous interfacial contacts that suppress electron leakage, facilitate Li^+^ transport, homogenize interfacial charge distribution, and regulate uniform Li deposition, while also contributing to stress accommodation and contact stability.

**Fig. 1:**
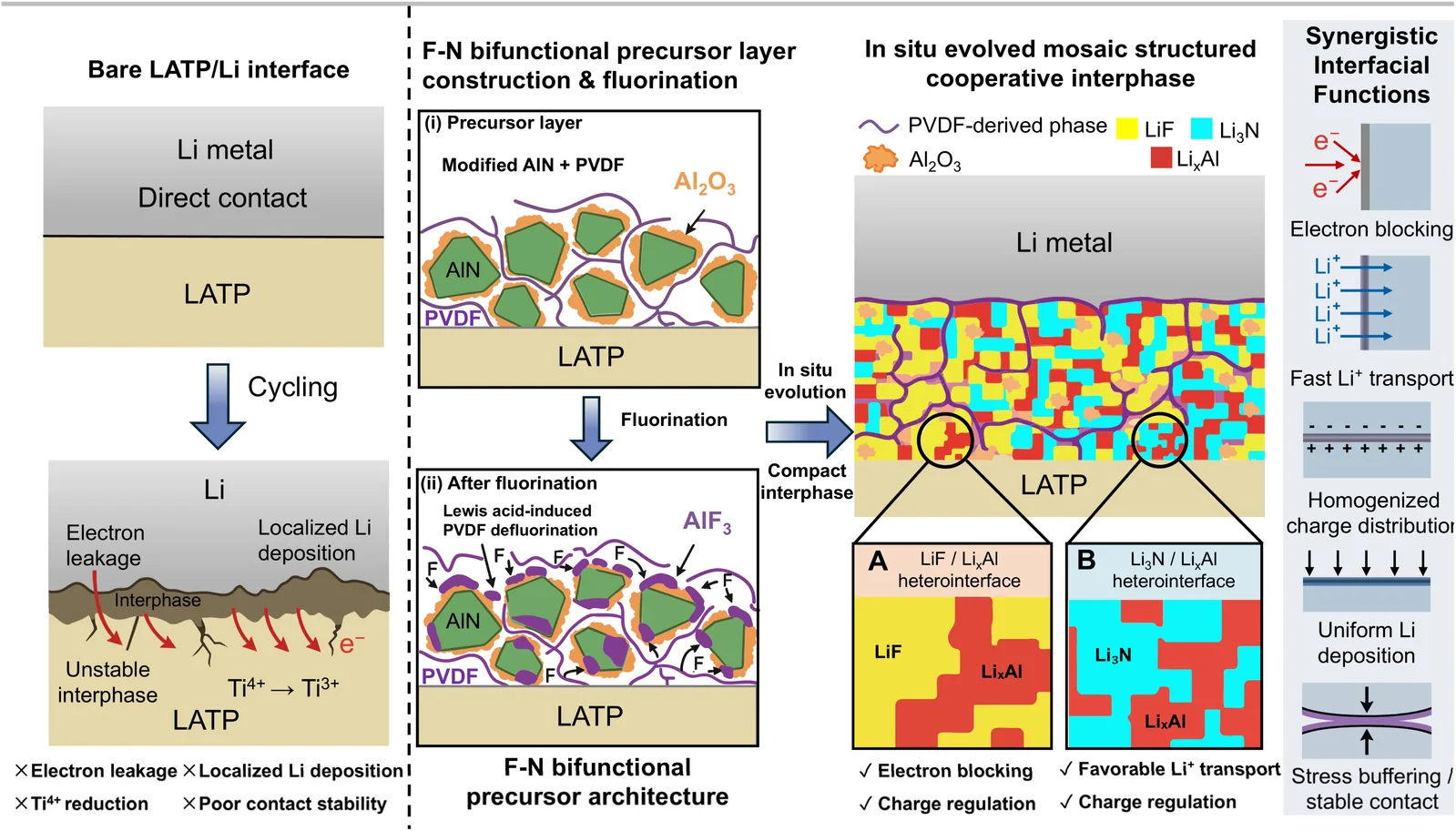
Schematic illustration of the instability of the bare LATP|Li interface, the construction and partial fluorination of the modified AlN/PVDF precursor layer, and its electrochemically driven *in situ* evolution into a mosaic-structured cooperative interphase.

XRD results show that MA largely retains the characteristic reflections of hexagonal AlN, while additional peaks assignable to Al_2_O_3_ emerge after treatment, indicating that the thermal process preserves the bulk AlN phase while introducing detectable oxide species on the particle surface (Fig. 2a). TEM analysis further confirms this partially oxidized feature: a surface-modified layer is tightly attached to the AlN core, with lattice spacings of 0.277 and 0.244 nm corresponding to the (021) plane of Al_2_O_3_ and the (002) plane of AlN, respectively, indicating close integration between the oxidized shell and the AlN matrix (Fig. 2b). The flocculent surface oxide layer on MA is further observed in the low-magnification TEM image (Fig. S1). The coexistence of Al, N, and O in elemental mapping further demonstrates that MA is a surface-oxidized AlN derivative rather than a fully oxidized product (Fig. 2c). This partially oxidized configuration is important because it preserves the AlN core while simultaneously introducing surface-active oxygen-containing sites, thereby creating a structural basis for subsequent chemical interaction with PVDF without eliminating the nitride framework. Meanwhile, the LATP pellet exhibits a dense ceramic microstructure with clear grain boundaries and negligible visible pores (Fig. S2), while electrochemical measurements confirm its high ionic conductivity of 7.2 × 10^−4^ S cm^−1^ and low electronic conductivity of 1.54 × 10^−9^ S cm^−1^ at 25 °C (Fig. S3). After introducing the PMA coating, LATP@PMA still retains the characteristic NASICON-type LATP phase, while additional reflections from MA become visible, indicating that the coating is successfully deposited on the LATP surface without disrupting the crystal structure of the substrate (Fig. S4).

**Fig. 2:**
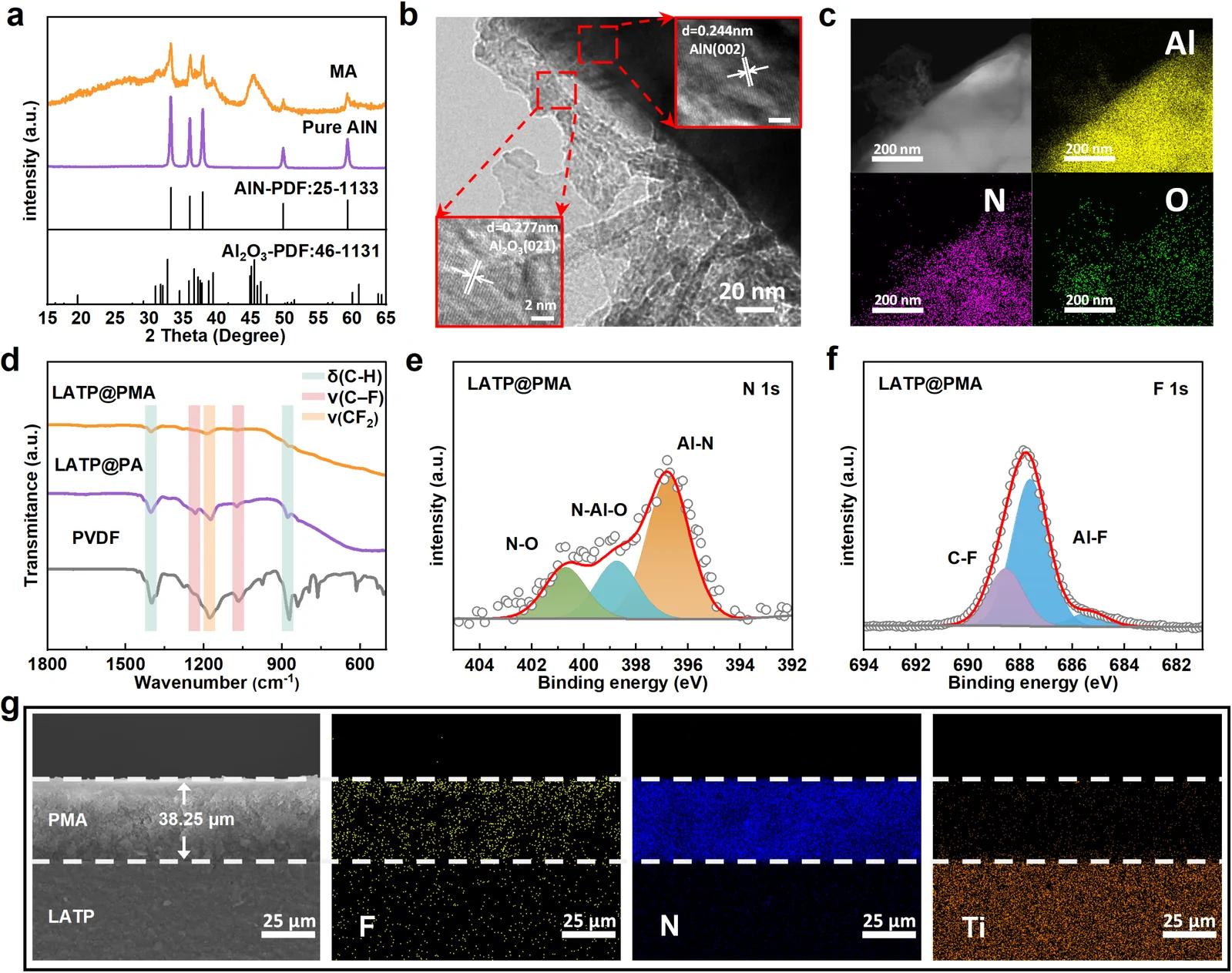
Structural and chemical characterization of MA and the PMA precursor coating on LATP. (a) XRD patterns of pure AlN and modified AlN (MA). (b) TEM image of MA, with the enlarged high-resolution TEM images showing lattice fringes corresponding to Al_2_O_3_ and AlN. (c) STEM-EDS elemental mappings of MA, showing the spatial distribution of Al, N, and O. (d) FTIR spectra of PVDF, LATP@PA, and LATP@PMA. High-resolution XPS spectra of PMA: (e) N 1s and (f) F 1s. (g) Cross-sectional SEM image and corresponding elemental mappings of LATP@PMA, showing the distributions of F, N, and Ti across the coating/electrolyte region.

To further clarify the chemical evolution during coating formation, FTIR analyses were carried out on PVDF, LATP@PA, and LATP@PMA. Compared with PVDF and LATP@PA, the characteristic –CF_2_– vibration bands (1073 and 1232 cm^−1^) and C–F vibration band (1173 cm^−1^) in LATP@PMA were markedly weakened or nearly disappeared, while the absorption peaks corresponding to C–H bending vibrations (1402 and 877 cm^−1^) were also attenuated, suggesting the occurrence of PVDF defluorination (Fig. 2d).^33,34^ The visible color change of the PMA precursor solution after heating further indicates chemical interaction between MA and PVDF during coating preparation, consistent with the defluorination behavior suggested by FTIR analysis (Fig. S5). Although PVDF defluorination is generally considered undesirable in polymer-based electrolytes, in the present design MA was introduced because its alumina-containing surface provides Lewis acid sites to induce this process, thereby enabling it to be harnessed as an *in situ* interfacial fluorination pathway during precursor-coating formation.^35–37^ XPS results provided further support for such chemical evolution. In the N 1s spectrum of PMA, Al–N (396.7 eV), N–Al–O (398.7 eV), and N–O (400.7 eV) species coexisted, indicating that the coating retains AlN structural units while introducing oxidized/oxynitridated active sites (Fig. 2e).^38^ In the F 1s spectrum, both C–F (688.5 eV) and Al–F (687.6 and 685.3 eV) signals were detected, indicating that part of the fluorine-containing species migrates from the polymer environment and is immobilized by the inorganic phase (Fig. 2f).^39,40^ In the Al 2p spectrum, the coexistence of Al–N (73.8 eV), Al–O (74.8 eV), and Al–F (76.1 eV) species further supported the involvement of partially oxidized AlN in PVDF defluorination and the *in situ* formation of Al–F species (Fig. S6).^38,39^ By contrast, only Al–N and C–F signals were observed for the PA coating, confirming that the key factor for initiating PVDF defluorination is the surface oxidation layer on AlN rather than pristine AlN itself (Fig. S7). This contrast between PA and PMA is critical because it indicates that the later interfacial differences are not caused simply by the presence of AlN, but by the modified precursor chemistry introduced through partial oxidation. The optical image of LATP@PMA shows a uniform and intact coating on the LATP pellet surface, suggesting good macroscopic coverage of the PMA layer (Fig. S8). Cross-sectional SEM and EDS mapping further confirmed that the PMA coating uniformly enveloped the LATP surface, forming a continuous and dense F/N-rich functional layer (Fig. 2g), in agreement with the intended structure.

Following the identification of a tightly coupled F–N bifunctional precursor coating, electrochemical measurements were further carried out to track its electrochemical conversion and evaluate the role of this precursor-layer strategy in interfacial evolution. As shown in Fig. 3a and b, the half-cell assembled with PMA as the cathode and Li metal as the anode exhibits a pronounced irreversible capacity in the first cycle, with an initial coulombic efficiency of 31.18%, indicating substantial irreversible electrochemical conversion of the PMA components during the initial discharge process (Fig. 3a).^29,41,42^ The corresponding d*Q*/d*V* profiles further resolve this conversion behavior: the reduction peaks centered at ∼1.6 and ∼1.5 V can be assigned to the conversion reactions of AlN and AlF_3_ with Li (AlN + 3Li^+^ + 3e^−^ → Al + Li_3_N; AlF_3_ + 3Li^+^ + 3e^−^ → Al + 3LiF), while the low-potential peak at ∼0.75 V corresponds to the alloying reaction between Al and Li (Al + *x*Li^+^ + *x*e^−^ → Li_*x*_Al).^43–45^ A distinct oxidation peak at ∼1.0 V in the subsequent charge process is most likely associated with the dealloying of Li_*x*_Al, indicating that Li_*x*_Al related species contribute a clear reversible component after the initial conversion, whereas the larger low-potential capacity is more reasonably regarded as a composite contribution involving the carbon/binder background and interfacial processes (Fig. 3b).^46^ The assignment of these characteristic features is further supported by the control electrodes, in which the blank electrode mainly reflects background electrolyte decomposition, whereas the PVDF/Al_2_O_3_ and PA controls correspond to the AlF_3_ related and AlN related reactions, respectively (Fig. S9–S11). These results indicate that the F, N, and Al containing species in the PMA precursor coating undergo coupled electrochemical conversion, thereby laying the foundation for the subsequent *in situ* construction of a mosaic-structured interphase.

**Fig. 3:**
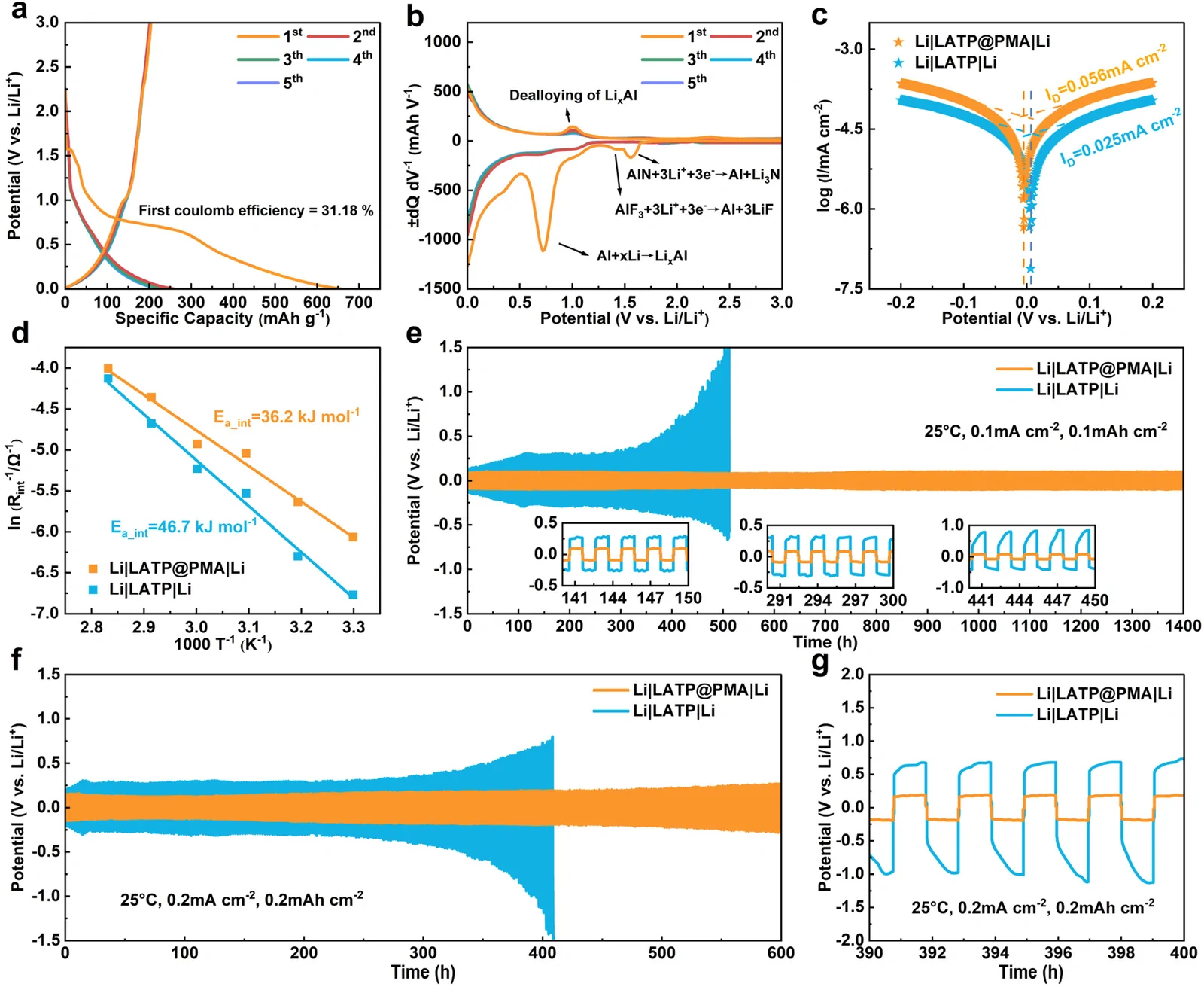
Electrochemical kinetics and Li plating/stripping stability of the PMA-modified LATP|Li interface. (a) The discharge and charge curves and (b) corresponding differential capacity curves of the PMA electrode. (c) Tafel plots of the Li|LATP@PMA|Li and Li|LATP|Li symmetric cells. (d) Arrhenius plots derived from electrochemical impedance spectroscopy (EIS) for the Li|LATP@PMA|Li and Li|LATP|Li interfaces. (e) Voltage–time curves of Li|LATP@PMA|Li and Li|LATP|Li symmetric cells at 0.1 mA cm^−2^/0.1 mA h cm^−2^. (f) Voltage–time and (g) magnified voltage–time curves of Li|LATP@PMA|Li and Li|LATP|Li symmetric cells at 0.2 mA cm^−2^/0.2 mA h cm^−2^.

To evaluate the influence of the conversion-derived interphase on interfacial reaction kinetics, symmetric cells based on Li|LATP@PMA|Li and Li|LATP|Li were further analyzed. After introducing the PMA precursor coating, the exchange current density of Li|LATP@PMA|Li becomes markedly higher than that of the unmodified counterpart, indicating improved interfacial charge-transfer kinetics (Fig. 3c). Meanwhile, the Arrhenius plots derived from electrochemical impedance spectroscopy show that the apparent activation energy of the Li|LATP@PMA|Li interface decreases from 46.7 to 36.2 kJ mol^−1^, suggesting that the conversion-derived interphase reduces the interfacial reaction barrier and facilitates Li^+^ transport (Fig. 3d).^47^ The corresponding temperature-dependent EIS spectra are provided in Fig. S12. To further evaluate interfacial Li^+^ transport, chronoamperometric polarization combined with EIS was performed (Fig. S13). The apparent Li^+^ transference number of Li|LATP@PMA|Li reaches 0.70, compared with 0.38 for Li|LATP|Li, further supporting more effective Li^+^ transfer across the PMA-modified interface. In addition, LSV measurements showed that the upper limit of the electrochemical stability window increased from approximately 4.24 V for LATP to 4.93 V for LATP@PMA, based on a current–density criterion of 0.03 mA cm^−2^ (Fig. S14). The interfacial current tolerance was further evaluated by stepwise critical current density measurements (Fig. S15). The Li|LATP@PMA|Li cell maintained regular Li plating/stripping responses up to 1.8 mA cm^−2^, whereas the Li|LATP|Li cell exhibited pronounced voltage instability at approximately 0.4 mA cm^−2^. Furthermore, in symmetric-cell cycling tests at 25 °C, Li|LATP@PMA|Li can cycle stably for more than 1400 h at 0.1 mA cm^−2^/0.1 mA h cm^−2^, while the unmodified Li|LATP|Li cell becomes unstable after around 450 h (Fig. 3e). At a higher current density of 0.2 mA cm^−2^/0.2 mA h cm^−2^, Li|LATP@PMA|Li still remains stable for over 600 h, whereas Li|LATP|Li shows a pronounced increase in polarization and eventually fails after approximately 400 h (Fig. 3f and g). Rate-dependent Li plating/stripping behavior was further evaluated under stepwise current densities from 0.05 mA cm^−2^/0.05 mA h cm^−2^ to 0.5 mA cm^−2^/0.5 mA h cm^−2^ (Fig. S16). The Li|LATP@PMA|Li cell maintained regular and reversible voltage responses throughout the test, and its polarization largely recovered when the current density returned to 0.05 mA cm^−2^/0.05 mA h cm^−2^. In contrast, the Li|LATP|Li cell exhibited pronounced voltage fluctuations and irregular low-voltage responses at 0.3 mA cm^−2^/0.3 mA h cm^−2^, indicative of soft-short-circuit-like behavior. These results indicate that the conversion-derived interphase effectively mitigates interfacial parasitic reactions and local deposition instability, thereby markedly improving the reversible Li plating/stripping stability of the LATP|Li interface.

To further verify the chemical composition of the interphase formed after electrochemical conversion of the precursor coating and its effect on the stability of the LATP|Li interface, XPS and morphological characterizations were carried out on the interfacial layers of LATP@PMA and bare LATP after 100 h of cycling. As shown in Fig. 4a–c, the N 1s, F 1s, and Al 2p spectra of cycled LATP@PMA exhibit pronounced compositional evolution, whereas the corresponding signals from bare LATP are much weaker, indicating that the PMA precursor coating continues to participate in interfacial reactions during cycling and dominates the chemical evolution of the interphase. Specifically, new Li–N (400.6 eV) and C–N (397.7 eV) components are observed in the N 1s spectrum of cycled LATP@PMA, with the Li–N signal attributable to Li_3_N formation, whereas the C–N component is likely associated with C–N bond formation involving trace solvent-derived species during the reduction process (Fig. 4a).^48^ In the F 1s spectra, the interphase on LATP@PMA is dominated by LiF (684.8 eV), whereas that on bare LATP is dominated by Li_*x*_PO_*y*_F_*z*_ (681.1 eV), suggesting that the bare LATP surface is mainly covered by a discontinuous and poorly stable interphase arising from decomposition of the trace wetting electrolyte (Fig. 4b).^48^ In the Al 2p spectrum of cycled LATP@PMA (Fig. 4c), prominent signals corresponding to Li_*x*_Al (71.2 eV) and metallic Al^0^ (72.7 eV) are clearly observed, whereas these signals are much weaker or nearly absent in bare LATP.^45^ A signal at 74.1 eV corresponding to Al–O species is also detected, consistent with aluminum oxide. Compared with the Al–O peak in the initial PMA coating (74.8 eV), the slight shift likely reflects minor changes in the local chemical environment or interfacial interactions during cycling.^49^ This stable oxide phase remains intact throughout cycling and contributes to increased interfacial density and structural integrity of the coating.^50^ These results further confirm that the PMA precursor coating undergoes continued chemical reconstruction during cycling to form a LiF, Li_3_N, and Li_*x*_Al containing cooperative interphase, thereby establishing the chemical and structural basis for stable LATP|Li interfacial regulation under demanding solid-state full-cell conditions.

**Fig. 4:**
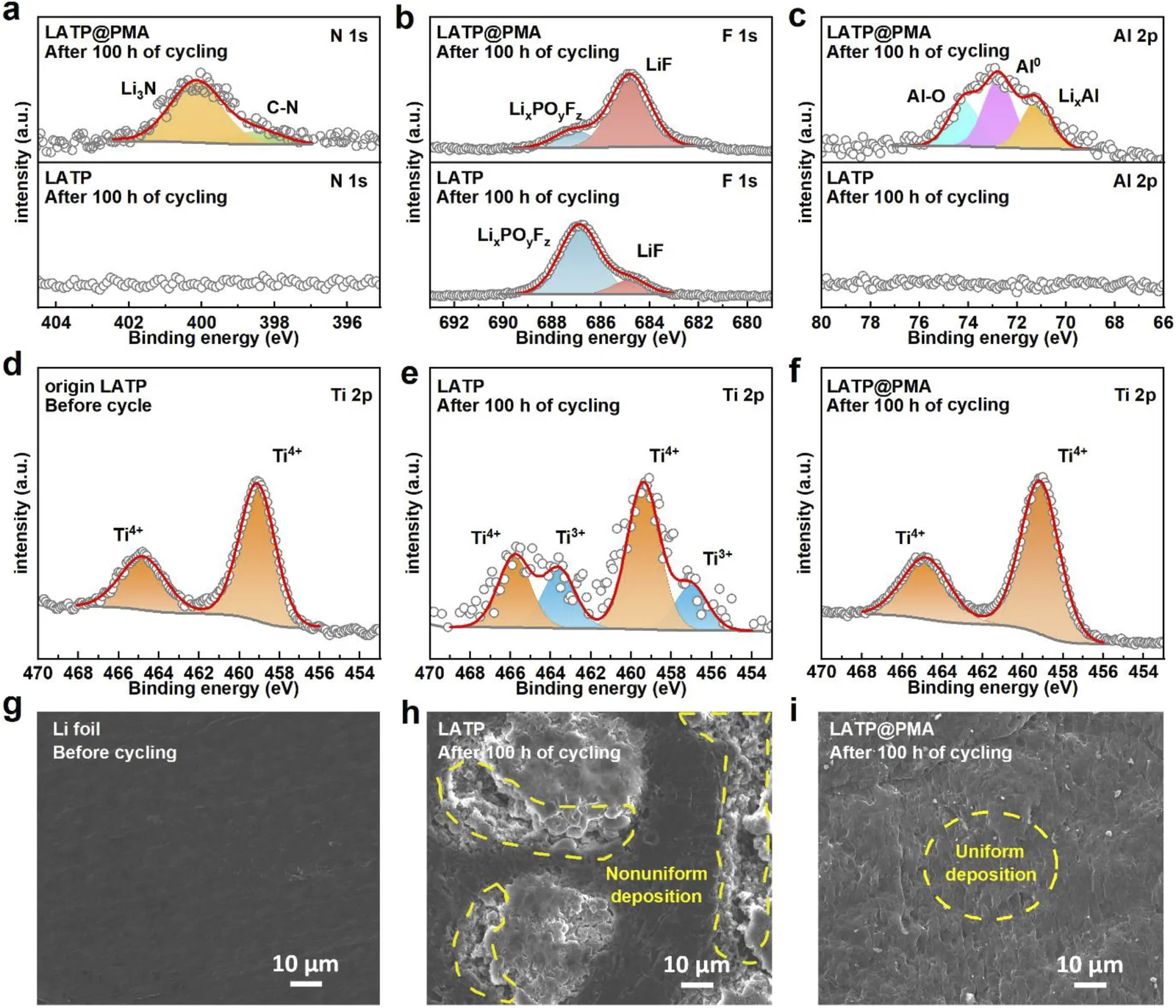
Chemical and morphological evidence for the stabilized LATP|Li interface enabled by the evolved PMA-derived interphase. High-resolution XPS spectra of the cycled interfacial layers, comparing LATP@PMA and bare LATP: (a) N 1s, (b) F 1s, and (c) Al 2p. High-resolution Ti 2p XPS spectra of LATP: (d) pristine LATP before cycling, (e) bare LATP after cycling, and (f) LATP@PMA after cycling. (g) SEM image of pristine Li foil before cycling. SEM images of Li foil after cycling against (h) bare LATP and (i) LATP@PMA.

To further verify that the constructed cooperative interphase can effectively suppress the reductive decomposition of LATP and improve the chemical compatibility of the LATP|Li interface, the Ti 2p spectra before and after cycling were comparatively analyzed. Pristine LATP exhibited only the characteristic Ti^4+^ signals (465.2 and 459.2 eV) before cycling (Fig. 4d), whereas after 100 h of cycling, the Ti 2p spectrum of bare LATP shifted toward lower binding energy and displayed an additional Ti^3+^ related component (463.2 and 458.3 eV), indicating that Ti^4+^ was reduced to Ti^3+^ upon cycling against Li metal and that LATP underwent interfacial reductive decomposition (Fig. 4e).^29^ In contrast, the Ti 2p spectrum of LATP@PMA after cycling remained dominated by Ti^4+^, with no obvious Ti^3+^ related signal and a much less pronounced peak shift, indicating that the evolved interphase effectively suppressed the reductive decomposition of LATP and thereby markedly improved the chemical stability of the LATP|Li interface (Fig. 4f).

The improved interfacial chemical stability was further reflected in the morphology of the Li metal after cycling. The pristine Li foil exhibited a smooth and dense surface before cycling (Fig. 4g). After cycling against bare LATP, the Li foil became rough and porous, showing pronounced nonuniform deposition (Fig. 4h). In contrast, the Li foil cycled against LATP@PMA maintained a relatively dense and smooth surface, without obvious dendritic or severely destabilized deposits (Fig. 4i). Taken together, the XPS and SEM results demonstrate that the PMA precursor coating, originating from PVDF-defluorination-enabled precursor fluorination, undergoes subsequent interfacial reconstruction during cycling to form a stable, continuous, and dense interphase enriched in LiF, Li_3_N, and Li_*x*_Al.

To further elucidate the spatial distribution of the key components in the evolved interphase and their interfacial interaction characteristics, TOF-SIMS depth profiling was conducted on the cycled LATP@PMA interphase. As shown in Fig. 5a, characteristic ionic fragments, including LiF^−^, LiN^−^, Li_2_Al^−^, LiAl^−^, and AlO_2_^−^, remained detectable throughout the sputtering process, whereas the PO_2_F^−^ signal was relatively weak, indicating that the cycled interphase is mainly enriched with LiF, Li_3_N related species, Li_*x*_Al, and Al–O species, consistent with the XPS results. The three-dimensional overlay further showed that these species are not confined to isolated regions, but instead exhibit extensive coexistence and overlap across the interphase (Fig. S17). Individual three-dimensional reconstructions further revealed that LiF^−^, LiN^−^, Li_2_Al^−^, and LiAl^−^ are continuously co-distributed throughout the interphase (Fig. 5b). In contrast, the AlO_2_^−^ and PO_2_F^−^ signals (Fig. S18), corresponding to Al_2_O_3_ and Li_*x*_PO_*y*_F_*z*_ species, respectively, show distinct characteristics: AlO_2_^−^ is mainly associated with a stable oxide phase that contributes to interfacial compactness and structural integrity,^51^ whereas the PO_2_F^−^ signal is sparse and mainly originates from decomposition of trace wetting electrolyte.^48^ Accordingly, neither Al_2_O_3_ nor Li_*x*_PO_*y*_F_*z*_ is considered to dominate the interfacial functionality.

**Fig. 5:**
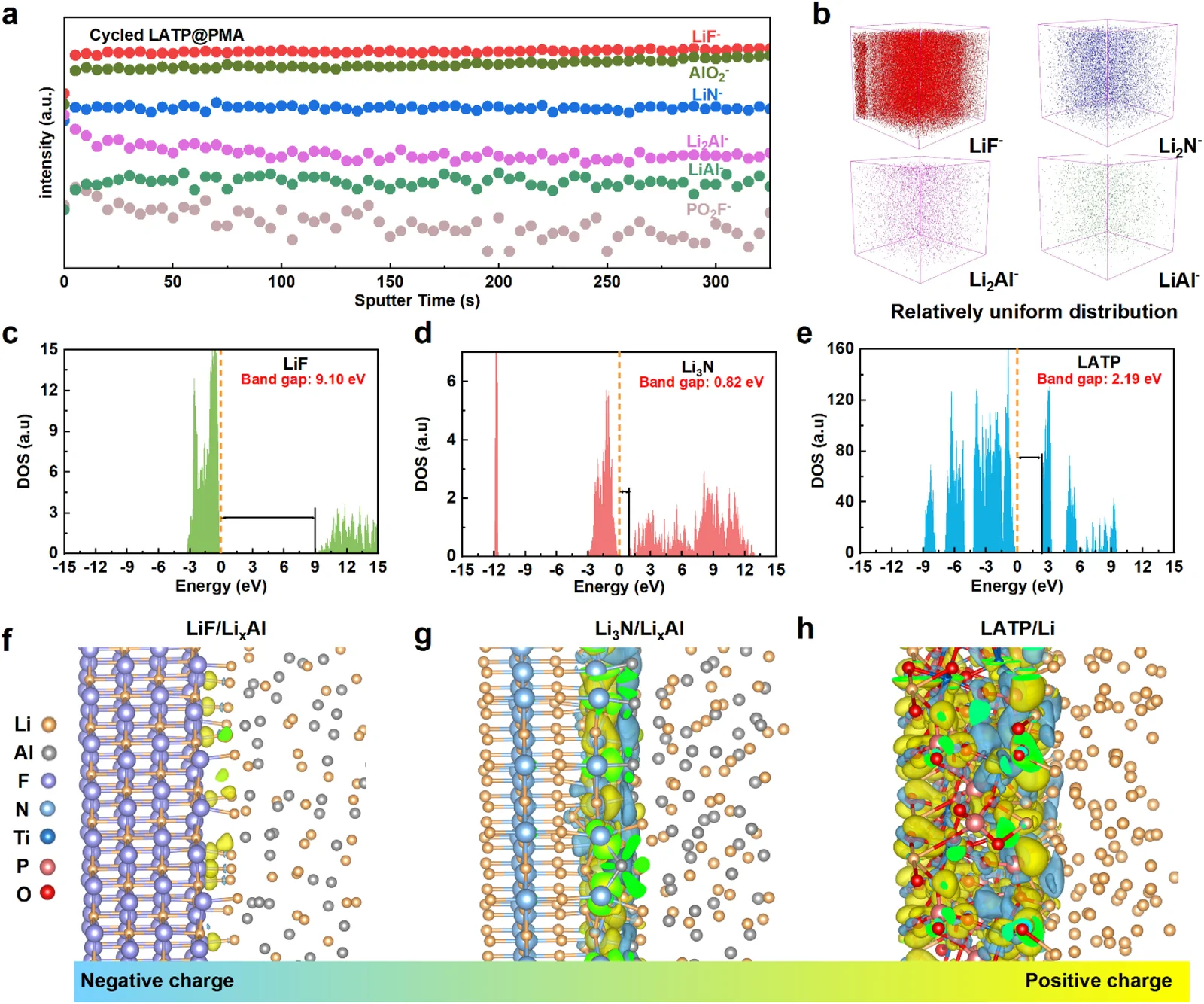
Spatial distribution and local electronic characteristics of the evolved mosaic-structured interphase on LATP@PMA. (a) TOF-SIMS depth profiles of characteristic secondary-ion fragments in the cycled LATP@PMA interphase, including LiF^−^, LiN^−^, Li_2_Al^−^, LiAl^−^, AlO_2_^−^, and PO_2_F^−^. (b) Three-dimensional spatial distributions of the corresponding characteristic species in the cycled interphase. Calculated density of states (DOS) of (c) LiF, (d) Li_3_N, and (e) LATP. Differential charge density plots of the (f) LiF/Li_*x*_Al, (g) Li_3_N/Li_*x*_Al, and (h) LATP|Li interfaces.

To gain further insight into the electronic characteristics of the evolved interphase, theoretical calculations were carried out on the representative components and heterointerfaces. The DOS results revealed that LiF possesses the widest bandgap among the three phases, consistent with its role as the principal electronically insulating component in the evolved interphase (Fig. 5c–e).^52^ By contrast, Li_3_N exhibited the narrowest bandgap, indicating that Li_3_N alone is unlikely to provide sufficient electron-blocking capability and therefore requires complementary coupling with LiF to achieve the desired interfacial functionality.^48^ In view of their spatial distribution, relative abundance, and functional roles within the evolved interphase, LiF/Li_*x*_Al and Li_3_N/Li_*x*_Al were selected as representative local heterointerfaces for further analysis, with the direct LATP|Li contact taken as a reference. Differential charge density calculations showed that charge redistribution at both LiF/Li_*x*_Al and Li_3_N/Li_*x*_Al is largely confined to the interfacial region, whereas the LATP|Li interface displays a more evident tendency for electron penetration toward the LATP side (Fig. 5f–h). Notably, LiF/Li_*x*_Al exhibited the most pronounced electron-confinement behavior, further supporting the primary electron-blocking role of LiF, while Li_3_N/Li_*x*_Al still shows more localized interfacial charge redistribution than the direct LATP|Li contact. Collectively, these results indicate that, by constructing a mosaic-structured cooperative interphase with spatially coordinated heterogeneous domains of LiF, Li_3_N related species, and Li_*x*_Al, the evolved PMA-derived interface realizes synergistic electron blocking, Li^+^ transport, and interfacial regulation, thereby stabilizing the reactive LATP|Li interface.

Following the above analysis of the spatial distribution and electronic-structure characteristics of the evolved interphase, LATP-based solid-state full cells were further assembled using LFP cathodes to evaluate the electrochemical performance. As shown in Fig. 6a, the EIS result of the activated cell in the first cycle showed that LFP|LATP@PMA|Li full cell exhibited a smaller impedance response, indicating that the precursor coating, after *in situ* evolution at the anode side, effectively improves interfacial transport and reduces the initial polarization in the full-cell configuration. Consistent with this, long-term cycling at 0.5C showed that LATP@PMA maintained a higher and more stable reversible capacity, delivering 141.8 mA h g^−1^ with a capacity retention of 96.62% after 800 cycles, whereas the unmodified counterpart exhibited obvious capacity decay after about 145 cycles, decreasing to 79.6 mA h g^−1^ (Fig. 6b). These results demonstrate that PMA interfacial modification not only reduces the initial interfacial resistance of the full cell, but also effectively retards interfacial degradation during cycling, thereby improving the long-term cycling stability of LATP-based full cells.

**Fig. 6:**
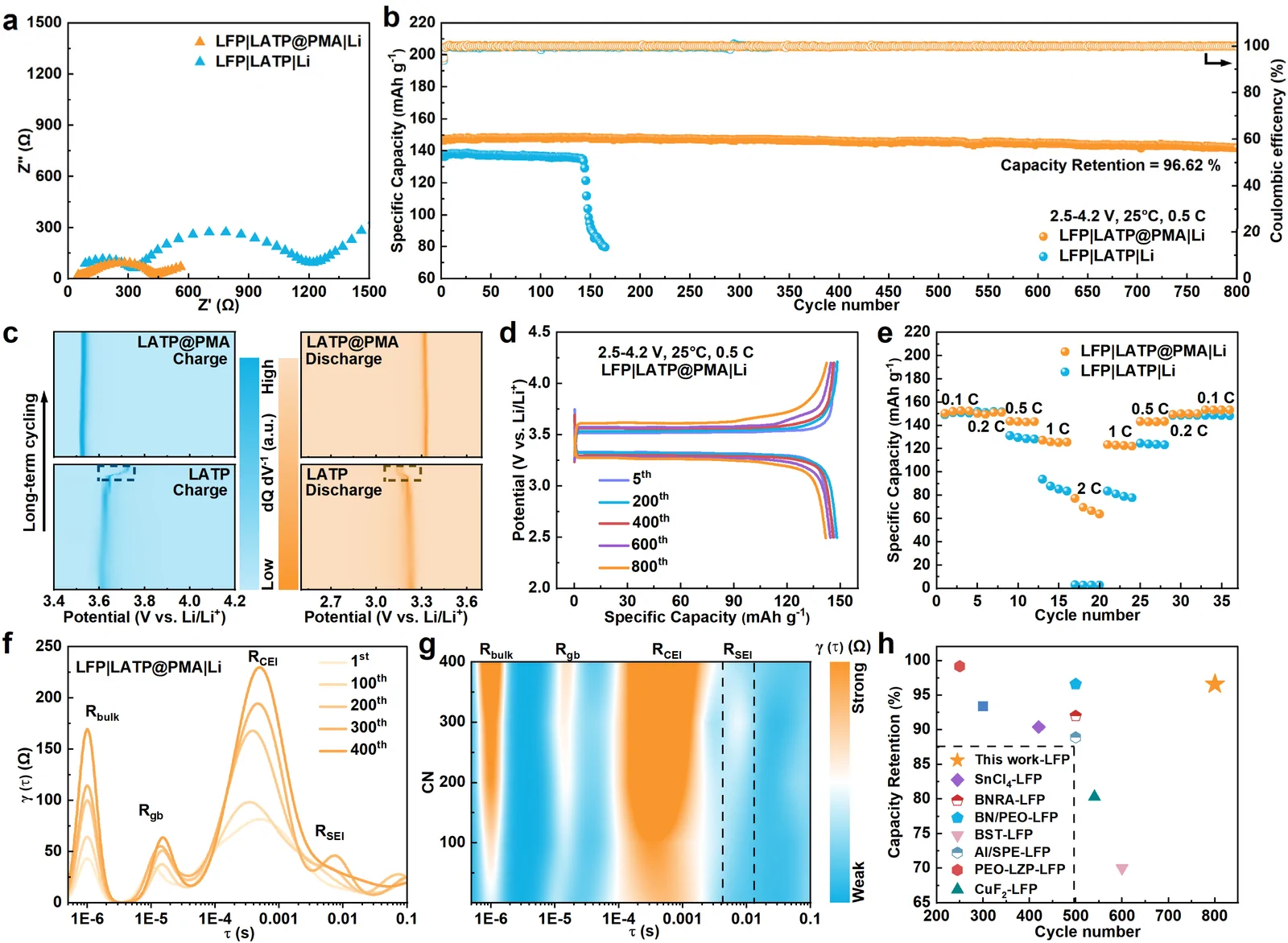
Electrochemical performance and interfacial kinetics of LATP-based LFP solid-state full cells with and without PMA modification. (a) Nyquist plots of activated LFP|LATP@PMA|Li and LFP|LATP|Li cells. (b) Long-term cycling performance at 25 °C and 0.5C within 2.5–4.2 V. (c) d*Q*/d*V* contour plots during long-term cycling. (d) Charge/discharge profiles of the LFP|LATP@PMA|Li full cell at selected cycle numbers. (e) Rate capability of the corresponding full cells. (f and g) EIS-derived DRT spectra and corresponding contour map of the LFP|LATP@PMA|Li cell during cycling. (h) Cycling-stability comparison with representative LATP-based interfacial-engineering systems.

The evolution of the electrode reaction during prolonged cycling was further visualized by the d*Q*/d*V* contour plots. As shown in Fig. 6c, the charge/discharge characteristic peaks of LFP|LATP@PMA|Li full cell remained relatively concentrated within the first 160 cycles, exhibiting only limited peak shift and broadening, which indicates that polarization growth is effectively suppressed during cycling. In contrast, the corresponding peaks of the unmodified cell gradually became diffuse and continuously shifted, reflecting progressive deterioration in the uniformity of the interfacial reaction. Correspondingly, the galvanostatic charge/discharge profiles of LATP@PMA at different cycle numbers retained similar plateau positions and a relatively small voltage hysteresis, and even after 800 cycles the plateau structure remained well preserved with only limited contraction (Fig. 6d). Rate capability measurements revealed that LATP@PMA delivered reversible capacities of 150.2, 150.1, 143.5, 127.3, and 77.2 mA h g^−1^ at 0.1, 0.2, 0.5, 1, and 2C, respectively. When the rate was switched back to 0.1C, the capacity recovered to 153.4 mA h g^−1^, demonstrating excellent rate response and capacity recovery that are markedly superior to those of the unmodified cell (Fig. 6e). These results indicate that the stable conversion interphase formed *via in situ* evolution at the anode side not only improves the initial interfacial contact, but also continuously alleviates polarization accumulation and maintains more stable electrochemical reaction kinetics during long-term cycling.

The EIS spectra of the LFP|LATP@PMA|Li full cell at different cycle numbers are shown in Fig. S19. To further clarify the origin of impedance evolution during cycling, the EIS data of LFP|LATP@PMA|Li full cell at different cycle numbers were analyzed by DRT. As shown in Fig. 6f, according to previous reports, the peaks with time constants (*τ* = *RC*) of approximately 10^−6^ s, 10^−5^ s, 10^−4^–10^−3^ s, and 10^−2^ s correspond to Li^+^ transport within the grain interior (*R*_bulk_), grain boundaries (*R*_gb_), the LFP|LATP interface (*R*_CEI_), and the Li|LATP interface (*R*_SEI_), respectively.^53,54^ Although the PMA coating provided clear protection to LATP at the anode side interface, prolonged Li stripping/plating at relatively high current densities still led to gradual increases in *R*_bulk_ and *R*_gb_, thereby contributing to the overall impedance growth. More importantly, the SEI associated resistance in the PMA modified full cell increased only slightly from 15 Ω in the first cycle to 22 Ω after 100 cycles, and remained below 46 Ω even after 400 cycles, whereas the corresponding SEI associated resistance in the unmodified full cell increased sharply from 90 Ω to 363 Ω within the first 100 cycles (Fig. S20). This pronounced contrast demonstrates that the PMA derived interphase effectively suppresses rapid impedance accumulation at the Li|LATP interface and thereby stabilizes the anode side reaction kinetics during prolonged cycling. By comparison, the CEI associated resistance in the PMA modified cell showed only a gradual increase during cycling, which is reasonable because no dedicated interfacial engineering was introduced at the cathode side and the trace wetting electrolyte could only partially improve the initial cathode/electrolyte contact, while the interface still remained essentially governed by solid-solid contact during prolonged cycling. Consistently, the contour map converted from the DRT spectra showed that the 10^−2^ s region changed only slightly during cycling, whereas the major impedance evolution was mainly concentrated in the 10^−4^–10^−3^ s range (Fig. 6g). Taken together, these results indicate that the cooperative interphase derived from the PMA precursor coating and its subsequent *in situ* evolution continuously stabilizes the LATP|Li interface and effectively retards rapid impedance buildup in LATP-based full cells during long-term cycling. Furthermore, the electrochemical performance of the LFP full cell in this work was compared with previously reported interfacial modification strategies for LATP-based systems (Fig. 6h).^15,25,29,55–58^ The LFP|LATP@PMA|Li full cell achieves a favorable balance between cycle life and capacity retention, maintaining 96.62% retention after 800 cycles and ranking among the better-performing interfacial-engineering systems reported to date.

After validating the effectiveness of the present interfacial modification strategy in LFP full cells, a higher-voltage NCM811 cathode was further employed to evaluate its applicability and interfacial regulation capability under more demanding conditions. As shown in Fig. 7a, the NCM811|LATP@PMA|Li cell maintained a higher and more stable reversible capacity during long-term cycling at 0.5C, delivering 153.2 mA h g^−1^ after 150 cycles with a capacity retention of 82.14%, whereas the unmodified counterpart underwent continuous capacity decay, dropping to 13 mA h g^−1^ after 150 cycles. Representative charge/discharge profiles of both cells at selected cycle numbers are provided in Fig. S21. This result indicates that the beneficial effect of the evolved interphase remains operative even under the more severe chemo-mechanical fluctuation associated with the NCM811 cathode.^22^ The clear advantage of LATP@PMA in the NCM811 system further suggests that, beyond interfacial electrochemical regulation, improving the ability of the full-cell interfacial system to accommodate stress fluctuation and maintain stable contact may also be important for stable cycling under harsher conditions.^59^ More broadly, the cycle-resolved d*Q*/d*V* contour maps show that the principal redox features of the NCM811|LATP@PMA|Li cell remained continuous and relatively concentrated during prolonged cycling (Fig. S22). By contrast, the corresponding features of the NCM811|LATP|Li cell progressively weakened and became diffuse, reflecting continuously increasing polarization and deterioration of the reaction uniformity. Further comparison with representative previously reported NCM811-based LATP solid-state systems showed that LATP@PMA achieves a favorable balance between initial specific capacity and long-term capacity retention, placing its overall performance among the better-performing systems of this class (Fig. 7b).^25,29,58,60,61^

**Fig. 7:**
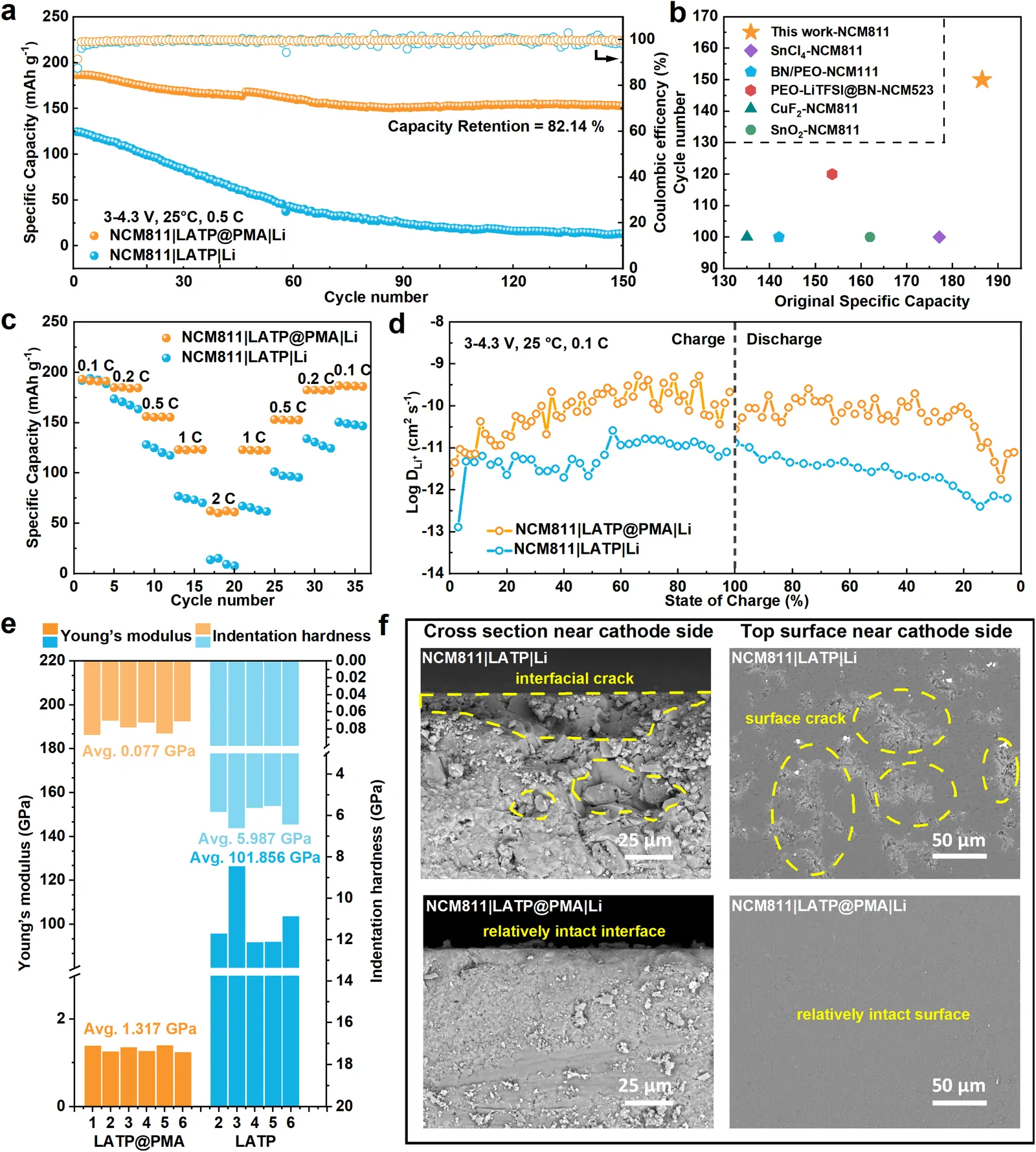
Electrochemical performance, mechanical properties, and post-activation structural integrity of NCM811-based LATP solid-state full cells. (a) Long-term cycling performance of NCM811|LATP@PMA|Li and NCM811|LATP|Li cells at 25 °C and 0.5C within 3.0–4.3 V. (b) Performance comparison with representative reported LATP-based interfacial-engineering systems. (c) Rate capability of the corresponding full cells. (d) Relationship between log *D*_Li^+^_ and state of charge derived from GITT measurements. (e) Young's modulus and indentation hardness of LATP@PMA and bare LATP determined by nanoindentation. (f) Cross-sectional and top-view SEM images near the cathode side of the recovered LATP pellets after two activation cycles at 0.1C.

Rate capability measurements further reveal the effectiveness of this interfacial regulation strategy under high-current conditions. As shown in Fig. 7c, the NCM811|LATP@PMA|Li cell delivered discharge capacities of 193.2, 184.9, 156.1, 123.2 and 62.1 mA h g^−1^ at 0.1, 0.2, 0.5, 1, and 2C, respectively, all markedly higher than those of the unmodified cell, which delivered 191.7, 173.5, 128.2, 76.8, and 13.7 mA h g^−1^ under the same conditions. When the rate returns to 0.1C, the modified cell recovered to 186.6 mA h g^−1^, demonstrating superior rate reversibility and interfacial recovery capability. GITT analysis further supports the improved kinetic behavior of the modified full cell, with the corresponding GITT curves provided in Fig. S23 and the derived Li^+^ diffusion behavior shown in Fig. 7d. These results suggest that the cooperative interphase derived from the PMA precursor coating and its subsequent *in situ* evolution helps maintain lower reaction polarization and more effective Li^+^ transport at the LATP|Li interface under high-rate conditions.

To directly assess the mechanical response introduced by the PMA coating, nanoindentation measurements were performed at spatially separated locations on LATP@PMA and bare LATP surfaces (Fig. S24). As shown in Fig. 7e, LATP@PMA exhibits a Young's modulus of 1.317 ± 0.070 GPa and an indentation hardness of 0.077 ± 0.008 GPa, substantially lower than the corresponding values of bare LATP (101.856 ± 14.508 GPa and 5.987 ± 0.482 GPa, respectively). This more compliant mechanical response provides a mechanical basis for accommodating local deformation and mitigating stress concentration at the interface.

To assess the post-activation structural response of the LATP electrolyte while ensuring a consistent electrochemical history, the PMA-modified and unmodified NCM811 full cells were subjected to two activation cycles at 0.1C prior to disassembly. This protocol was selected to limit the cumulative contribution of parasitic reactions at the LATP|Li interface, thereby enabling a more direct comparison of cathode-side structural integrity. The corresponding charge/discharge profiles (Fig. S25), which document the electrochemical history before disassembly, show that the PMA-modified cell exhibited a slightly higher discharge capacity and lower polarization than the unmodified cell during the two-cycle protocol. The cathode-side cross section of the unmodified cell subsequently exhibited a pronounced interfacial crack, whereas the modified cell retained a comparatively intact interface. The top surface near the cathode side further revealed multiple surface cracks in the unmodified cell, whereas the modified cell maintained a comparatively intact surface with much less obvious damage (Fig. 7f). These observations are consistent with the superior cycling stability of the modified NCM811 full cell and suggest that the PMA derived cooperative interphase is beneficial for the full cell interfacial system to better tolerate chemo-mechanical fluctuation under demanding conditions, thereby mitigating crack propagation and structural deterioration of the brittle LATP electrolyte.^22^ These results further support the potential of this interfacial strategy for the future development of LATP-based solid-state batteries.

## Conclusions

In conclusion, we developed a simple modified AlN/PVDF coating strategy to stabilize the reactive LATP|Li-metal interface in solid-state batteries. In this design, modified AlN induces PVDF defluorination and establishes an F–N bifunctional inorganic filler framework within the PVDF matrix, which subsequently reacts *in situ* with Li metal to form an F–N bifunctional mosaic-structured interphase composed of coordinated LiF/Li_*x*_Al and Li_3_N/Li_*x*_Al heterogeneous microdomains. This evolved interphase enables electron blocking, Li^+^ transport, charge homogenization, and regulated Li deposition, while also improving stress accommodation and contact stability during cycling. Accordingly, the modified interface delivers excellent long-term stability in LFP solid-state full cells, achieving 141.8 mA h g^−1^ after 800 cycles with 96.62% capacity retention at 0.5C. The robustness of this interfacial design is further confirmed in the more demanding NCM811 solid-state full cells, which retain 82.14% capacity after 150 cycles at 0.5C. These findings establish spontaneous interphase evolution as an effective strategy for stabilizing the LATP|Li interface and provide an important interfacial foundation for the future development of high-energy-density solid-state batteries, alongside further reductions in solid-electrolyte thickness and advances in multilayer cell fabrication.

## Author contributions

Haijie Lin: experimental work, writing – original draft, writing – review and editing; Xiang Xie: some experimental data analysis; Fenghua Zheng: some experimental data analysis; Wei Liu: some experimental data analysis; Xinyou He: some experimental data analysis; Zhiming Xiao: TEM data analysis; Wei Xu: TEM data analysis; Fenghua Ding: some batteries assembly work; Xinghui Liang: supervision, writing – review and editing; Baishan Chen: writing – review and editing, funding acquisition; Lei Ming: writing – review and editing, funding acquisition; Zhang Lin: some experimental data analysis; Xing Ou: supervision, writing – review and editing, funding acquisition. All the authors discussed the experimental results.

## Conflicts of interest

There are no conflicts to declare.

## Supplementary Material

SC-017-D6SC04429H-s001

## Data Availability

The data supporting the findings of this study are available within the article and its supplementary information (SI). Supplementary information: experimental details, supplementary figures, electrochemical analysis, characterization data. See DOI: https://doi.org/10.1039/d6sc04429h.
