# Supplementary material for: A spontaneously evolving multifunctional interphase enables durable cycling in solid-state lithium metal batteries
Source: Chem Sci. 2026 Aug 5;17(38):18200–12. doi: 10.1039/d6sc04429h (PMC13458448; doi:10.1039/d6sc04429h)
Supplement: Supplementary file 1 [file SC-017-D6SC04429H-s001.pdf]

Supplementary Information (SI) for Chemical Science.
This journal is © The Royal Society of Chemistry 2026

Supporting Information

A Spontaneously Evolving Multifunctional Interphase Enables Durable Cycling in Solid-State Lithium Metal Batteries

Haijie Lin,[a] Xiang Xie,[a] Fenghua Zheng,[b] Wei Liu,[a] Xinyou He,[a] Zhiming Xiao,[a] Wei Xu,[a,c] Fenghua Ding,[a] Xinghui Liang,[a,*] Baishan Chen,[d,*] Lei Ming,[a,*] Zhang Lin,[a] Xing Ou [a,*]

[a] Engineering Research Center of the Ministry of Education for Advanced Battery Materials, School of Metallurgy and Environment, Central South University, Changsha, 410083, P.R. China

[b] Guangxi Key Laboratory of Low Carbon Energy Materials, School of Chemistry and Pharmaceutical Sciences, Guangxi Normal University, Guilin, 541004, PR China

[c] Quzhou Huayou Cobalt New Mat. Co. Ltd., Quzhou, 324000, P.R. China.

[d] Institute for Advanced Study, Central South University, Changsha 410083, China

Experimental Section

Preparation of LATP Pellets

$Li_{1.3}Al_{0.3}Ti_{1.7}(PO_4)_3$ (LATP) was synthesized via a conventional solid-state route. Li_2CO_3 (99%, Aladdin), Al_2O_3 (99.99%, Aladdin), TiO_2 (99.8%, Macklin), and $NH_4H_2PO_4$ (99%, Macklin) were weighed according to the stoichiometric ratio. An additional 5 wt% Li_2CO_3 was added to compensate for lithium volatilization during high-temperature treatment. The precursors were ground at 400 rpm for 7 h and calcined in air at 800 °C for 5 h to obtain LATP powder. To reduce the particle size and enhance sintering, the LATP powder was ball-milled with 1 wt% Li_3PO_4 in isopropanol at 500 rpm for 7 h. The resulting slurry was dried at 70 °C for 8 h. The dried powder was then uniaxially pressed into pellets (diameter: 15 mm) using 1 wt% polyvinyl alcohol (PVA) aqueous solution as the binder under 22 MPa for 10 min. The green pellets were embedded in LATP powder and sintered in air at 850 °C for 6 h with a heating rate of 2 °C min^{-1} to obtain dense LATP pellets. Finally, the pellet surfaces were sequentially polished with sandpapers of different grit sizes until a smooth finish was achieved.

Preparation of PMA layer

AlN powder (99.9%, Macklin) was sintered at 950 °C for 6 h to obtain partially surface-oxidized AlN (denoted as modified AlN). The modified AlN powder and PVDF were mixed at a mass ratio of 9:1 in N-methyl-2-pyrrolidone (NMP) to form a mixed solution with a concentration of 50 mg mL^{-1}. The solution was stirred at 450 rpm for 1 h at room temperature. Subsequently, 100 μL of the mixed solution was drop-cast onto

the surface of LATP pellets, followed by drying at 90 °C for 4 h to obtain PMA-coated LATP pellets (LATP@PMA). For comparison, pristine AlN powder and PVDF (9:1 by mass) were processed using the same procedure to prepare PA-coated LATP pellets (LATP@PA).

Characterizations

The crystal structures of the obtained samples were analyzed on a Rigaku Ultima IV X-ray diffractometer equipped with a Cu Kα radiation source operated at 40 kV and 40 mA. The XRD data were recorded within a 2θ interval of 10 °–65 °. The morphology of LATP pellets and lithium anodes, including both surface and cross-sectional features, was observed by field-emission scanning electron microscopy using a JEOL JSM-7500F instrument, and the related elemental distributions were examined by EDS. Surface chemical compositions and oxidation states were further probed using X-ray photoelectron spectroscopy on a PHI Quantera II system, Japan. STEM observations, together with EDS elemental mapping, were performed at Central South University on a JEOL JEM-ARM200CF microscope operated at 200 kV and fitted with a Schottky cold-field emission gun. FTIR spectra were collected with a Thermo Scientific iS50 spectrometer. After cycling, the chemical constituents and their spatial profiles at the PMA-modified interfaces were investigated by time-of-flight secondary ion mass spectrometry.

Nanoindentation measurements were performed on LATP@PMA and bare LATP surfaces using a KLA nanoindentation system. Maximum loads of 2 mN and 5 mN were applied to LATP@PMA and bare LATP, respectively. The lower load applied to

LATP@PMA was selected to avoid excessive penetration into the compliant coating and to minimize the contribution from the underlying LATP substrate. The resulting average maximum indentation depth was 1.165±0.049 μm, corresponding to approximately 3–4% of the coating thickness. For bare LATP, the higher load provided a sufficiently resolved unloading response for reliable analysis, with an average maximum indentation depth of 0.176±0.009 μm. Six spatially separated locations were initially selected on each sample. Young's modulus and indentation hardness were determined from the unloading segments using the Oliver–Pharr method.[1] All six measurements on LATP@PMA yielded valid results. For bare LATP, the first indentation did not yield a valid Oliver–Pharr fitting result and was excluded from the statistical analysis; the remaining five measurements were used.

Electrochemical Tests

For ionic conductivity measurements, LATP pellets were sputtered with gold as ion-blocking electrodes to form an Au|LATP|Au configuration. To assemble the Li/PMA, Li/PA and Li/PVDF+Al_2O_3 batteries, lithium foil was used as the anode, 1 M $LiPF_6$ in DEC/EC/EMC (1:1:1, v/v/v) served as the liquid electrolyte, and the PMA/PA/PVDF+Al_2O_3 electrode was used as the cathode. The PMA/PA/PVDF+Al_2O_3 cathode was prepared by drop-casting 80 μL of a 50 mg mL^{-1} PMA/PA/PVDF+Al_2O_3 solution onto Cu foil pre-coated with a conductive carbon black/PVDF layer (carbon black: PVDF = 1:1, w/w). To assemble lithium symmetric batteries, LATP@PMA (or bare LATP) pellets were sandwiched between two lithium foils. For full-cell fabrication, the cathode slurry was obtained by mixing the active cathode material,

either LFP or NCM811, with conductive carbon black and PVDF in NMP at a mass ratio of 8:1:1. The resulting suspension was coated onto Al foil, with the active material loading controlled at 1.8–2.0 mg cm^{-2}. After drying at 90 °C for 12 h, the electrodes were cut into circular disks. In the assembled cells, LFP or NCM811 served as the cathode, lithium metal foil was used as the anode, and LATP@PMA or pristine LATP acted as the solid electrolyte. To promote Li^+ transfer across the electrode/electrolyte interfaces, a small amount of liquid electrolyte, 1 M $LiPF_6$ in DEC/EC/EMC with a volume ratio of 1:1:1, was introduced at 3 μL cm^{-2}. All cells were finally assembled as CR2025 coin-type batteries in an argon-filled glovebox.

Electrochemical impedance spectroscopy was conducted to evaluate the ionic conductivity of LATP pellets and the interfacial resistance of both symmetric and full cells. The measurements were performed on a CHI614C electrochemical workstation within the frequency range of 1 MHz to 1 Hz, using a perturbation amplitude of 0.01 V. The ionic conductivity was determined using the following equation:

$$\sigma_i = \frac{L}{RS}$$

Where σ_i is the ionic conductivity (S cm^{-1}), $L$is the thickness of the solid electrolyte disc (cm), $R$is the total resistance (Ω), and S is the measurement area (cm^2).

The activation energy ($E_{a,int}$) at the solid–liquid interface was calculated using an Equation. In the experiment, the assembled lithium symmetric batteries were subjected to impedance spectroscopy over a controlled temperature range, and measurements were taken only after complete thermal stabilization at each target temperature. The obtained EIS data were then fitted with an equivalent circuit to determine the charge-

transfer resistance (Rct). The activation energy for interfacial migration was obtained by plotting and fitting the Arrhenius relationship using the corresponding equation.

$$\frac{1}{R_{ct}} = Aexp(-\frac{E_{a\_int}}{RT})$$

Tafel plots were obtained using lithium symmetric batteries at a fixed sweep rate of 0.1 mV s^{-1} over a voltage range from −0.2 to 0.2 V.

The apparent Li^+ transference number was evaluated using chronoamperometric polarization combined with electrochemical impedance spectroscopy before and after polarization. Li|LATP@PMA|Li and Li|LATP|Li symmetric cells were polarized under a constant DC voltage of 10 mV, and the current was recorded until a quasi-steady state was reached. The apparent Li^+ transference number was calculated according to the Bruce–Vincent–Evans equation:

$$t_{Li^+} = \frac{I_s(\Delta V - I_0R_0)}{I_0(\Delta V - I_sR_s)}$$

where I_0 and I_s are the initial and steady-state currents, respectively, ΔV is the applied polarization voltage, and R_0 and R_s are the interfacial resistances obtained from the impedance spectra before and after polarization, respectively.

Linear sweep voltammetry was performed using SS|LATP|Li and SS|LATP@PMA|Li cells, in which the stainless-steel electrode replaced the cathode while the remaining cell configuration and interfacial wetting conditions were kept unchanged. For the SS|LATP@PMA|Li cell, the PMA-coated surface faced the Li electrode. The cells were scanned from 2.5 to 6.0 V versus Li/Li^+ at a scan rate of 1 mV s^{-1}. The upper electrochemical stability limit was determined using a current-density criterion of 0.03 mA cm^{-2}.

For critical current density measurements, the current density and corresponding areal capacity were increased stepwise from 0.1 mA cm^{-2}/0.1 mAh cm^{-2} to 2.0 mA cm^{-}

^{2}/2.0 mAh cm^{-2} in increments of 0.1 mA cm^{-2}/0.1 mAh cm^{-2}, with each Li stripping or plating half-cycle lasting for 1 h.

Rate-performance measurements were conducted using Li|LATP@PMA|Li and Li|LATP|Li symmetric cells at 25 °C. The current density/areal capacity was sequentially increased from 0.05 mA cm^{-2}/0.05 mAh cm^{-2} to 0.1 mA cm^{-2}/0.1 mAh cm^{-2}, 0.2 mA cm^{-2}/0.2 mAh cm^{-2}, 0.3 mA cm^{-2}/0.3 mAh cm^{-2}, 0.4 mA cm^{-2}/0.4 mAh cm^{-2}, and 0.5 mA cm^{-2}/0.5 mAh cm^{-2}, followed by a return to 0.05 mA cm^{-2}/0.05 mAh cm^{-2}. Each Li plating or stripping half-cycle was maintained for 1 h.

Open-source MATLAB script-based software, Distribution of Relaxation Times (DRT) Tools, was used to calculate DRT from the impedance data.[2] The DRT calculation is based on Tikhonov regularization and discretization of continuous functions. The DRT Tools package incorporates both linear functions and radial basis functions (RBFs).

All long-term cycling tests of lithium symmetric batteries and full batteries were conducted on a LAND constant-temperature test system at 25 °C. To evaluate the stability against lithium, the lithium symmetric batteries were cycled under conditions of 1 h stripping and 1 h plating at constant current densities/capacities of 0.1 mA cm^{-2}/0.1 mAh cm^{-2} or 0.2 mA cm^{-2}/0.2 mAh cm^{-2}, with cut-off voltages set at −5 and 5 V. For full-battery tests, the voltage ranges were set to 2.5–4.2 V for Li//LFP batteries (1 C = 170 mA g^{-1}) and 3.0–4.3 V for Li//NCM811 batteries (1 C = 180 mA g^{-1}).

DFT calculations

All density functional theory (DFT) computations were performed with the Vienna Ab Initio Simulation Package (VASP).[3,4] The exchange–correlation energy was described under the generalized gradient approximation (GGA) using the PBE functional.[5] Core–valence interactions were modeled with the projected augmented wave (PAW) formalism,[6,7] while the valence electronic states were represented by a plane-wave basis set with an energy cutoff of 400 eV. Partial occupation of the Kohn–Sham states was treated using Gaussian smearing with a width of 0.05 eV. The self-consistent electronic calculation was stopped when the change in total energy fell below 10^{-5} eV. Geometry optimization was regarded as complete once the residual atomic forces were smaller than 0.02 eV/Å. Long-range dispersion effects were taken into account through Grimme's DFT-D3 correction scheme.[8]

The optimized equilibrium lattice parameters were a = 3.396 Å for BCC-Li, a = 4.010 Å for NaCl-type LiF, and a = 6.228 Å for cubic LiAl. For hexagonal Li3N, the optimized lattice constants were a = 3.502 Å and c = 3.720 Å. The hexagonal $LiTi_2(PO_4)_3$ unit cell was optimized with lattice parameters of a = 8.623 Å and c = 21.082 Å. Based on these optimized structures, three heterojunction slab models were constructed.

Model 1 corresponds to a LiAl(111)/Li_3N(001) heterojunction. The LiAl(111) slab was built with p(2×2) periodicity along the X and Y directions and contained two stoichiometric layers along the Z direction. The Li_3N(001) slab adopted p(5×5) periodicity in the X and Y directions and also consisted of two stoichiometric layers along the Z direction. A vacuum region of 15 Å was introduced along the Z direction

for the whole slab. This model contained 214 Li atoms, 64 Al atoms, and 50 N atoms. During geometry optimization, only the Γ point was used for Brillouin-zone sampling. The bottom stoichiometric layer of the $Li_3N(001)$ slab was kept fixed, whereas all other atoms were fully relaxed.

Model 2 represents the LiAl(111)/LiF(100) heterojunction. The LiAl(111) component was constructed with a $(2\times3\sqrt{3})$ in-plane periodicity and two stoichiometric layers along the Z direction, while the LiF(100) component was modeled using a p(3×8) periodicity in the X and Y directions and two stoichiometric layers along the Z direction. A 15 Å vacuum layer was added along the Z direction. The resulting model consisted of 144 Li atoms, 48 Al atoms, and 96 F atoms. For structural relaxation, a 2×1×1 k-point mesh was employed to sample the Brillouin zone. The bottom stoichiometric layer of the LiF(100) slab was constrained, and the remaining atoms were allowed to relax.

Model 3 was constructed as a $Li(110)/LiTi_2(PO_4)_3(012)$ heterojunction. The Li(110) slab had p(5×5) periodicity in the X and Y directions and three atomic layers along the Z direction. The $LiTi_2(PO_4)_3(012)$ slab was modeled with p(2×2) periodicity in the X and Y directions and two stoichiometric layers along the Z direction. A vacuum spacing of 15 Å was introduced along the Z direction. This model contained 162 Li atoms, 24 Ti atoms, 36 P atoms, and 144 O atoms. During the optimization, Γ-point sampling was adopted for the Brillouin zone. The bottom stoichiometric layer of the $LiTi_2(PO_4)_3(012)$ slab was fixed, while all other atoms were relaxed.

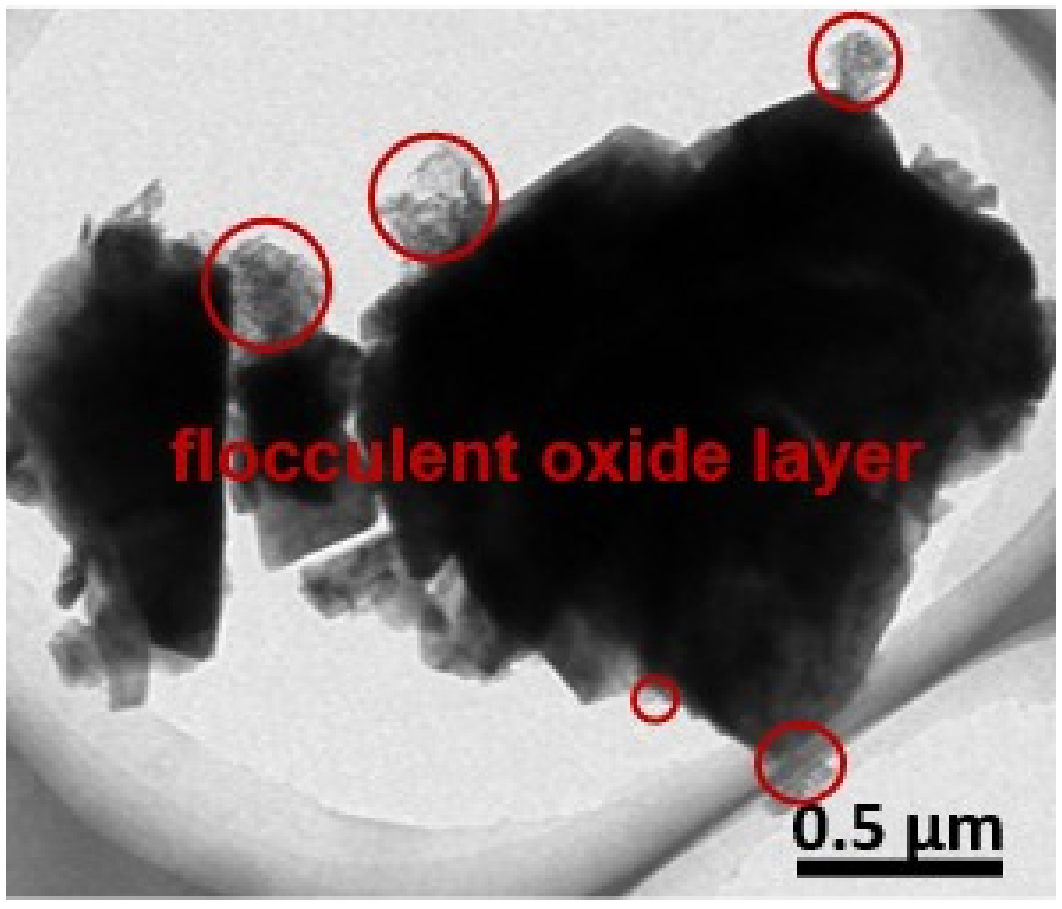

Fig. S1. TEM image of MA.

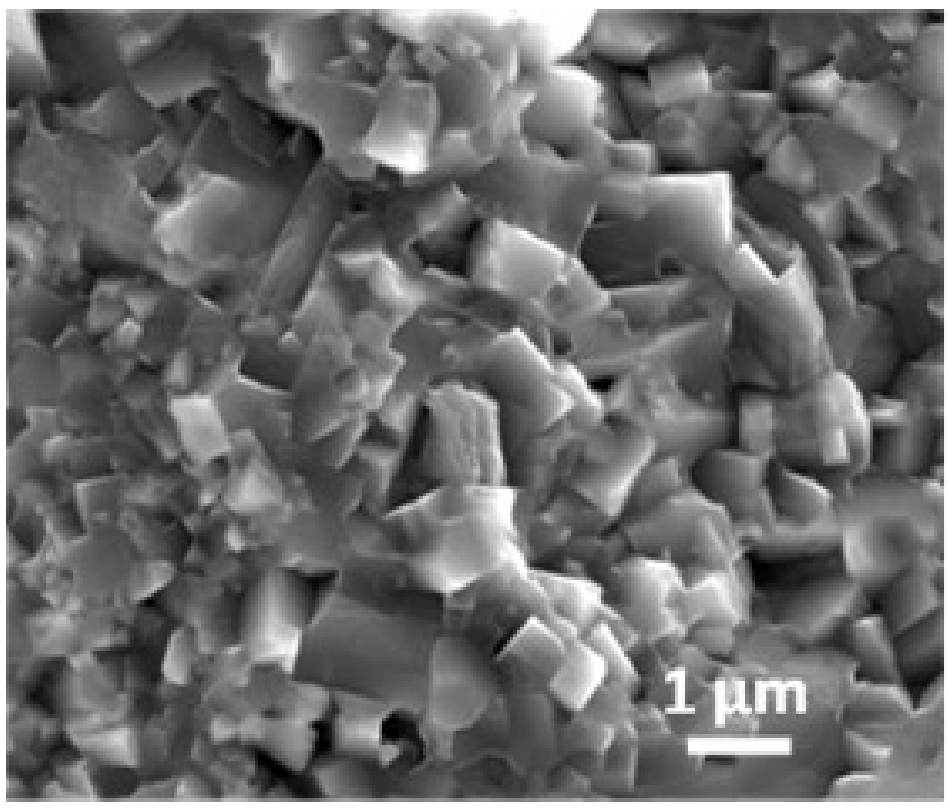

Fig. S2. SEM image of the cross-section of the LATP pellet.

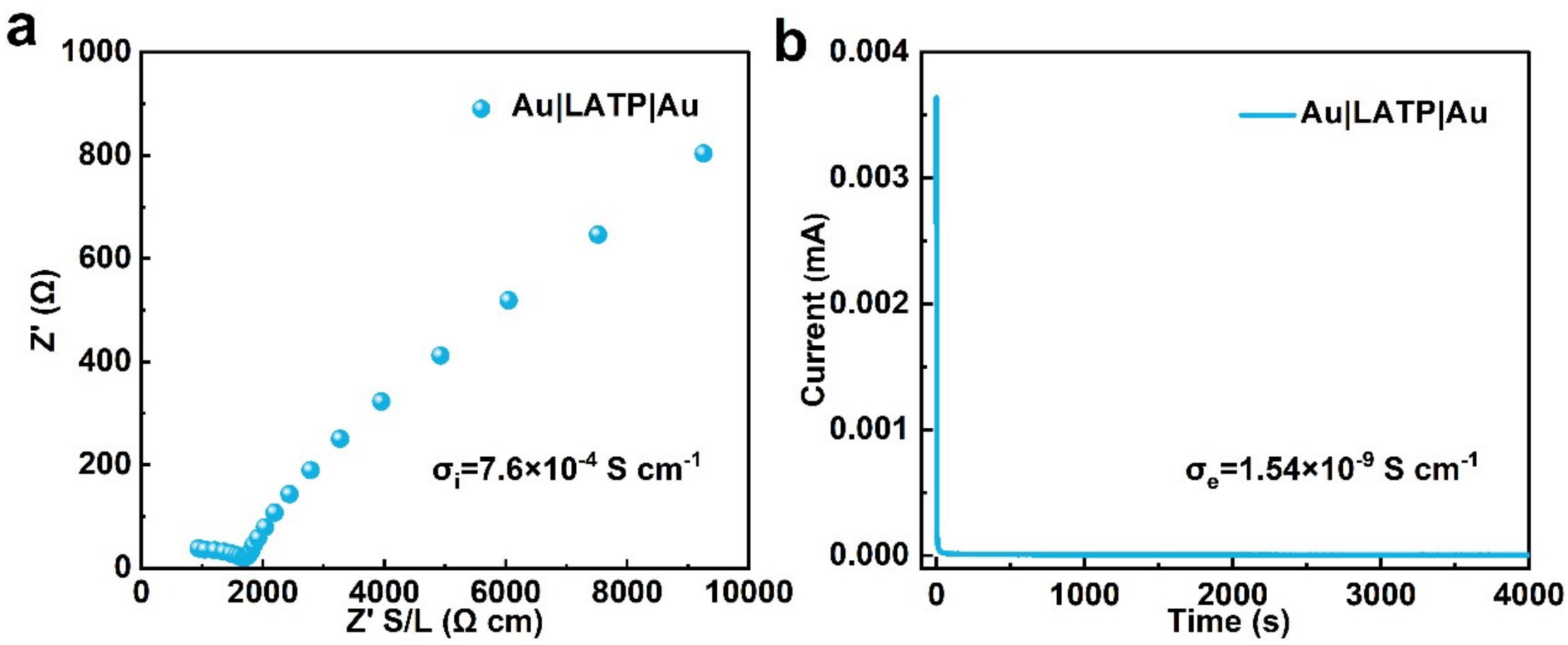

Fig. S3. a) Nyquist plots of Au|LATP|Au symmetric cells at room temperature; b) DC polarization plot of Au|LATP|Au at room temperature.

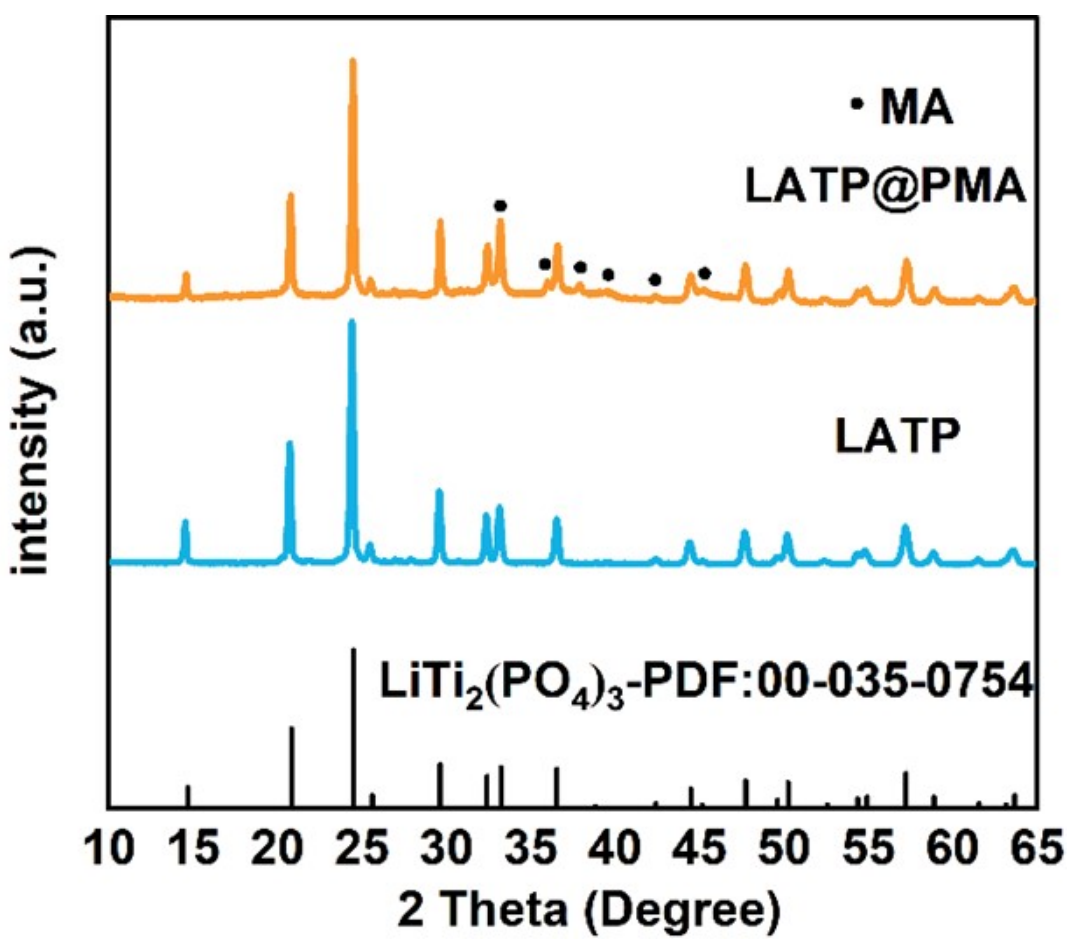

Fig. S4. XRD patterns of LATP and LATP@PMA.

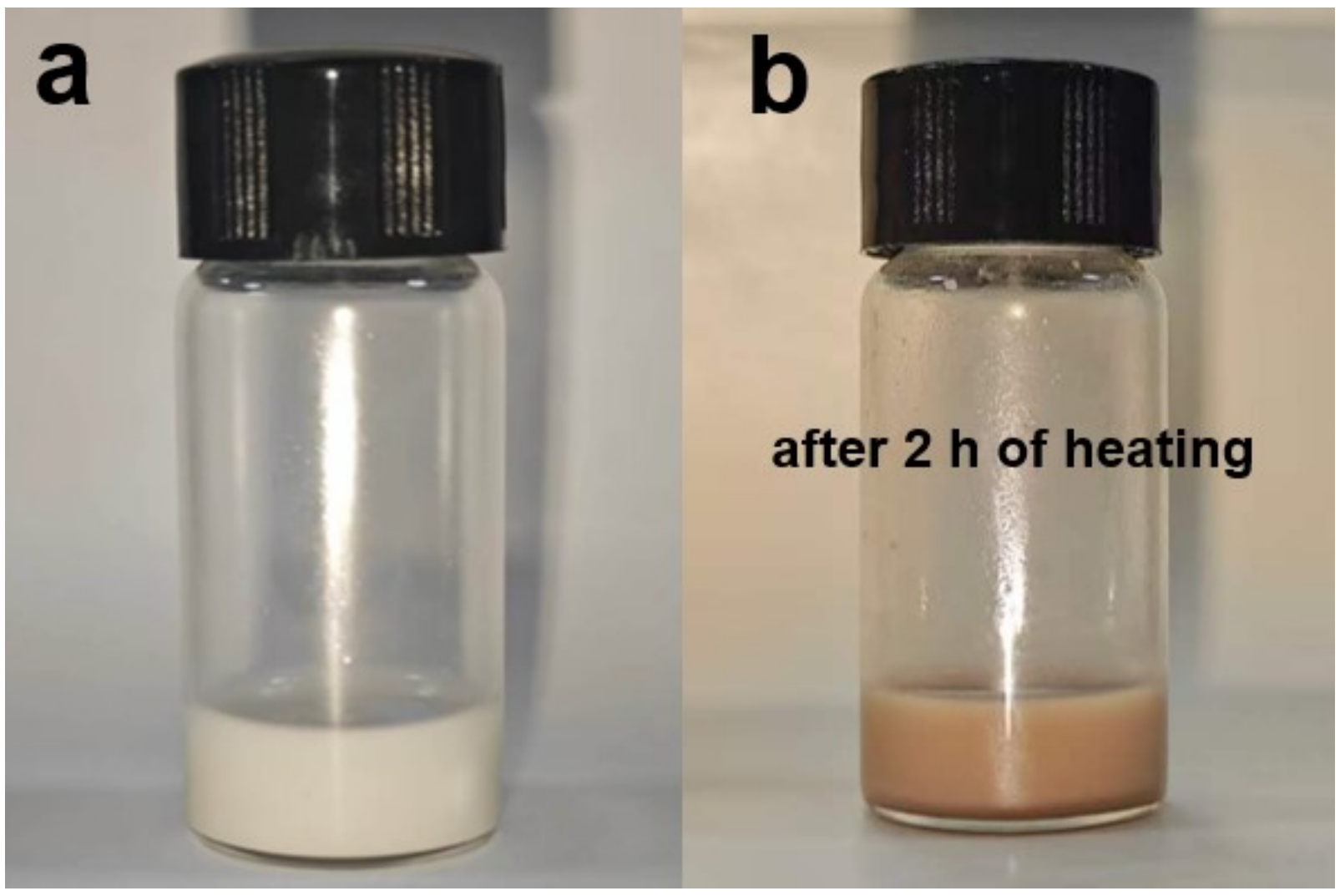

Fig. S5. a) Optical image of the PMA solution and b) optical image of the PMA solution after heating for 2 h.

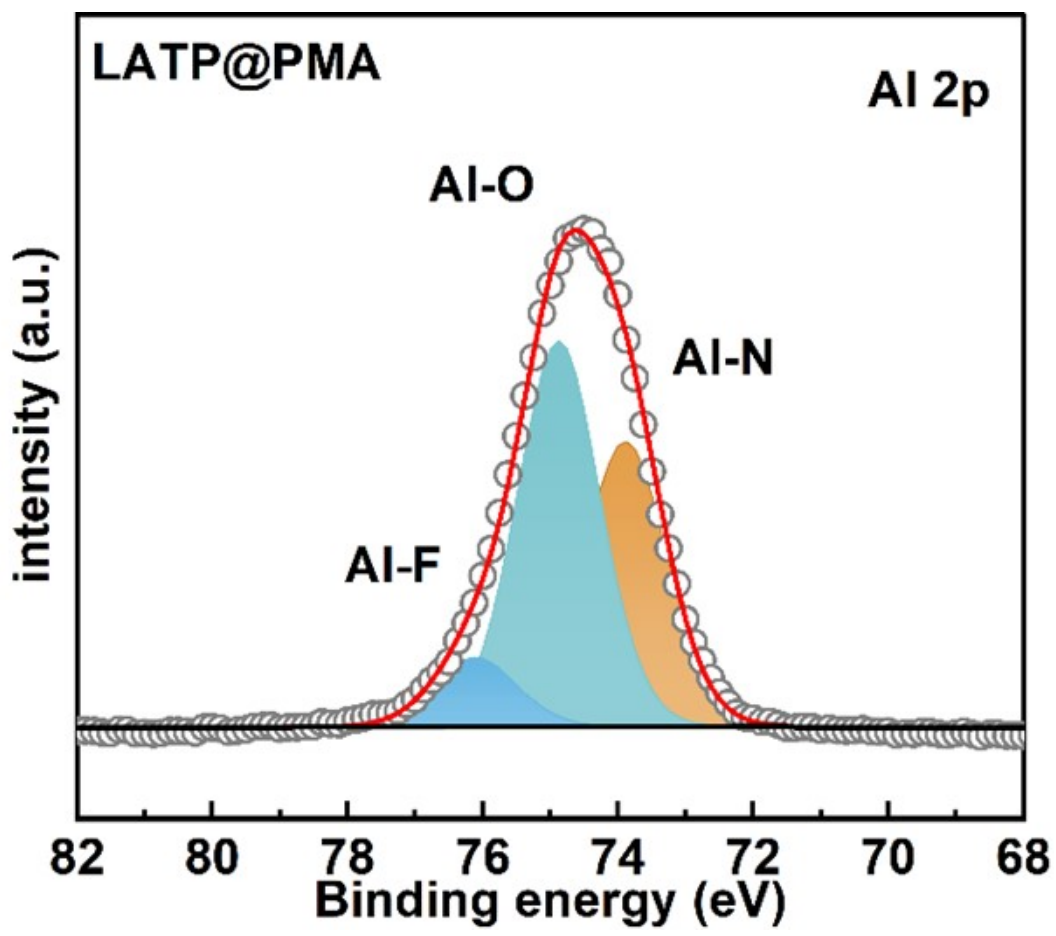

Fig. S6. High-resolution XPS spectra of PMA Al 2p.

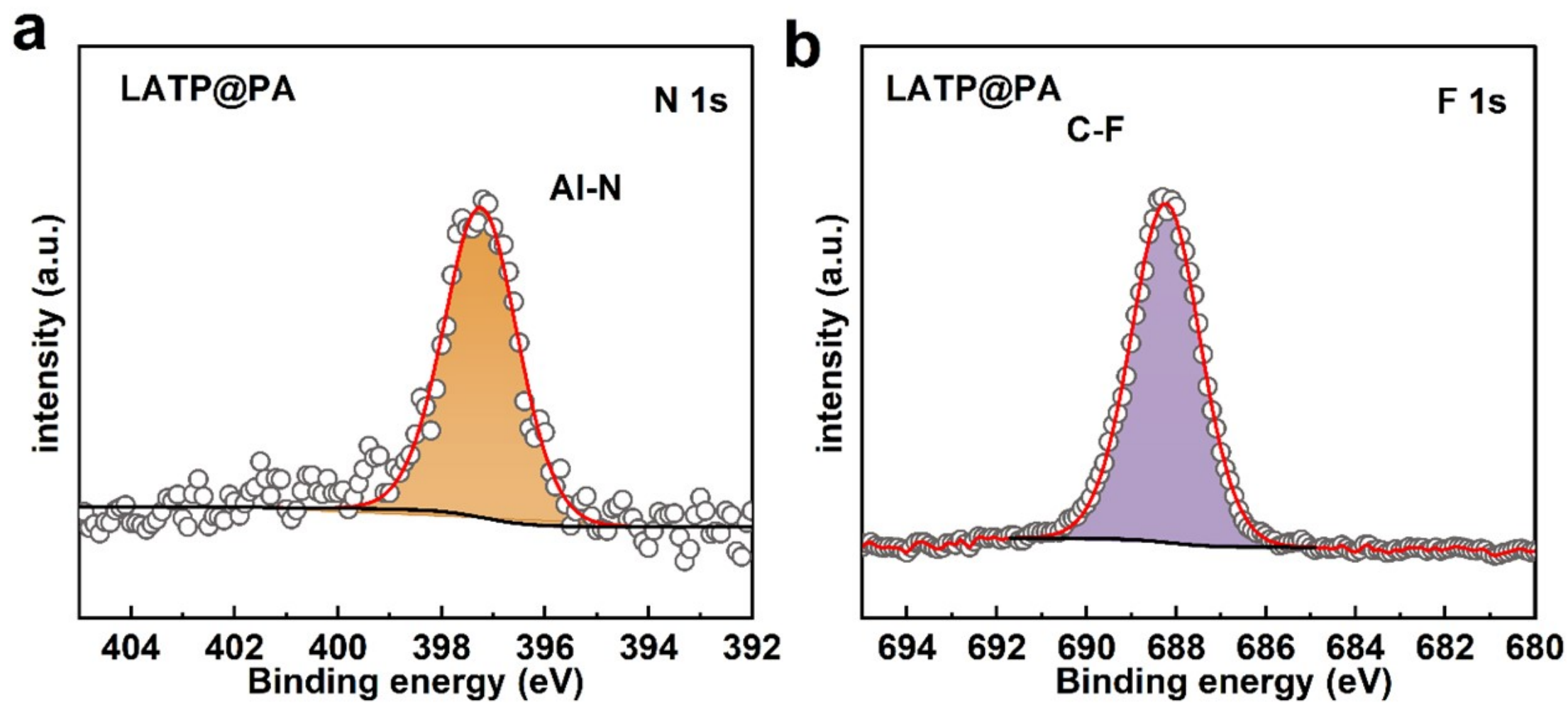

Fig. S7. a) High-resolution XPS spectra of PA N 1s; b) High-resolution XPS spectra of PA F 1s.

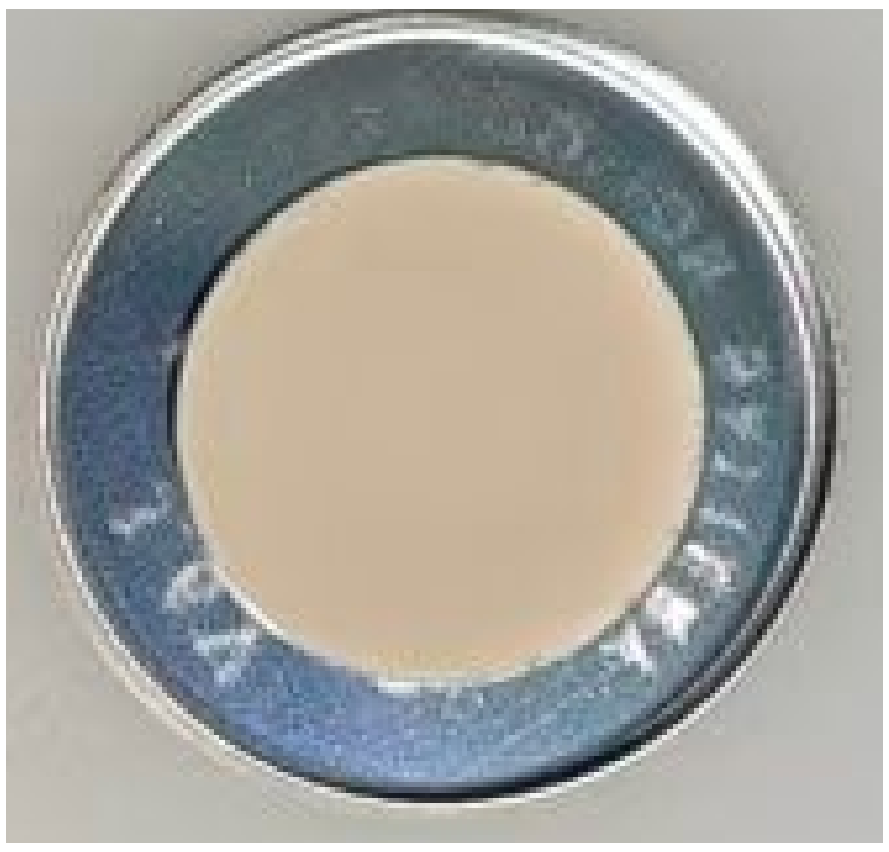

Fig. S8. Optical image of LATP@PMA.

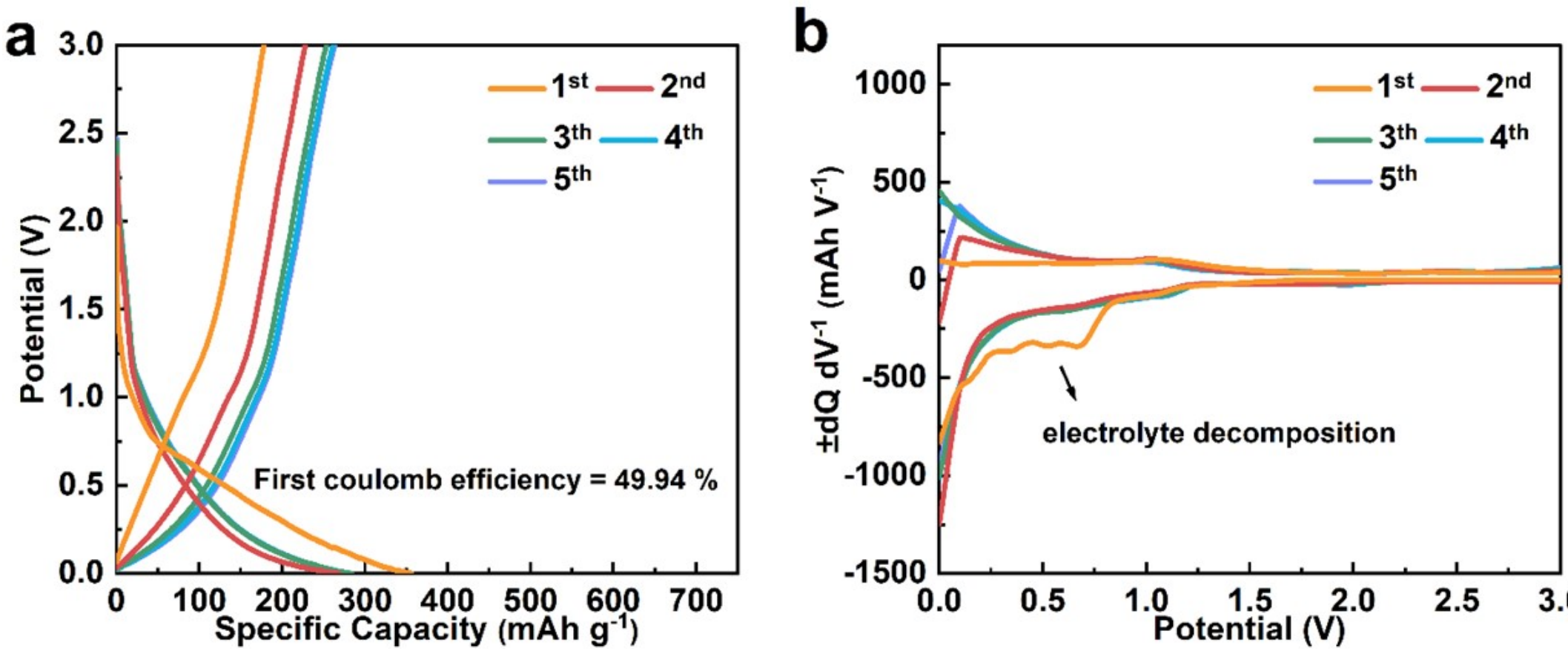

Fig. S9. a) The discharge and charge curves and b) corresponding differential capacity curves of blank electrode.

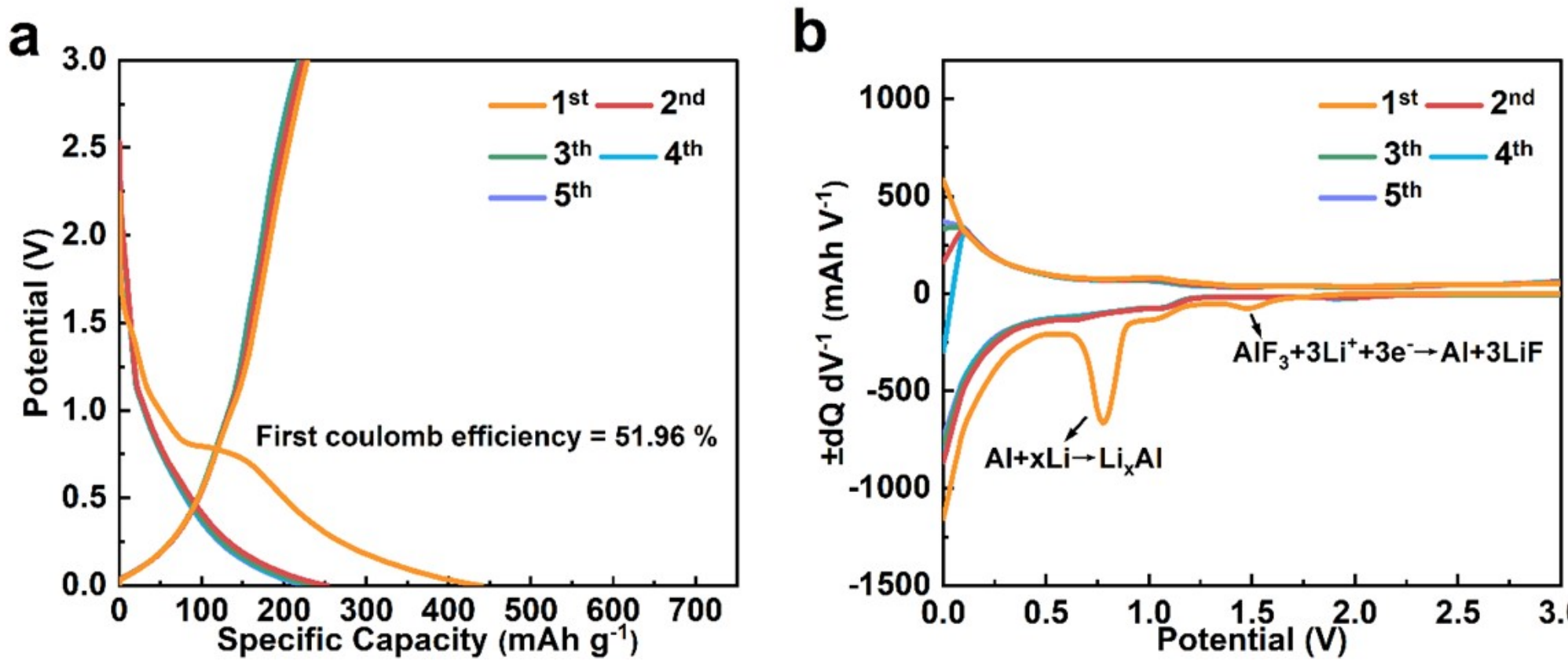

Fig. S10. a) The discharge and charge curves and b) corresponding differential capacity curves of PVDF+Al_2O_3 electrode.

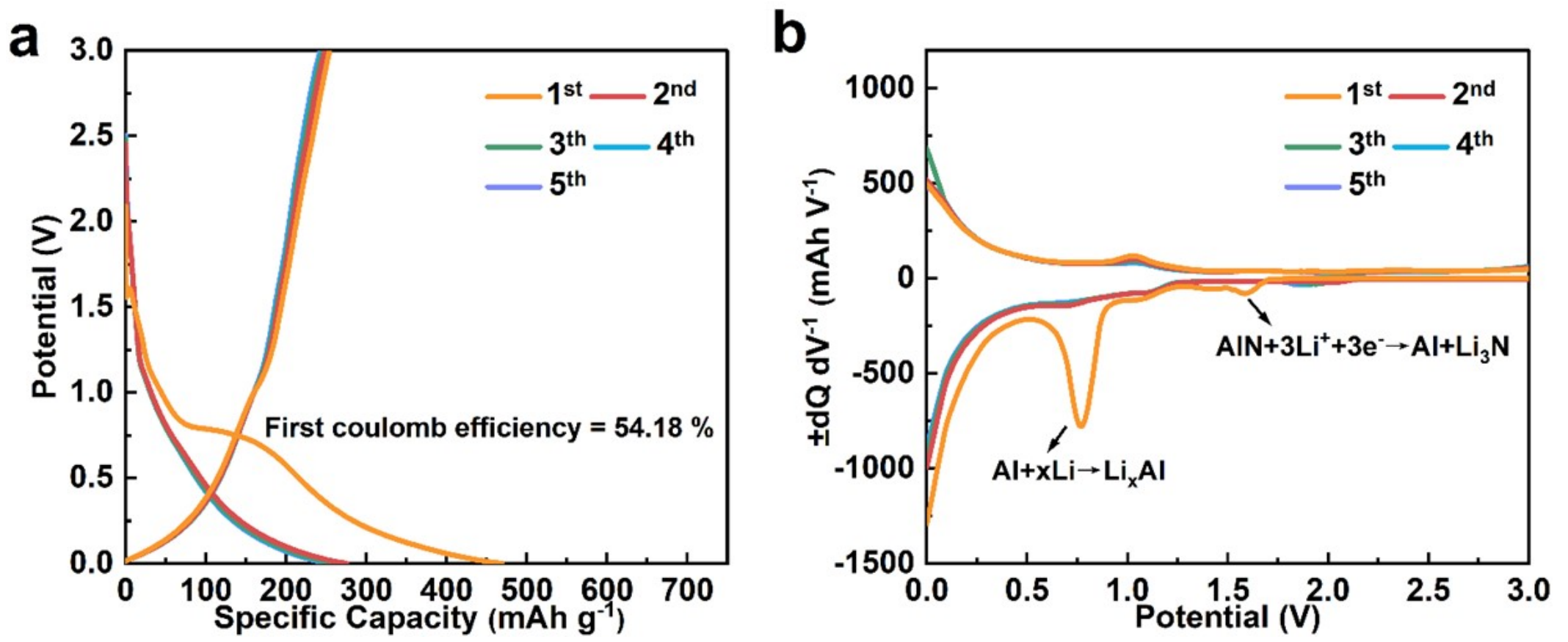

Fig. S11. a) The discharge and charge curves and b) corresponding differential capacity curves of PA electrode.

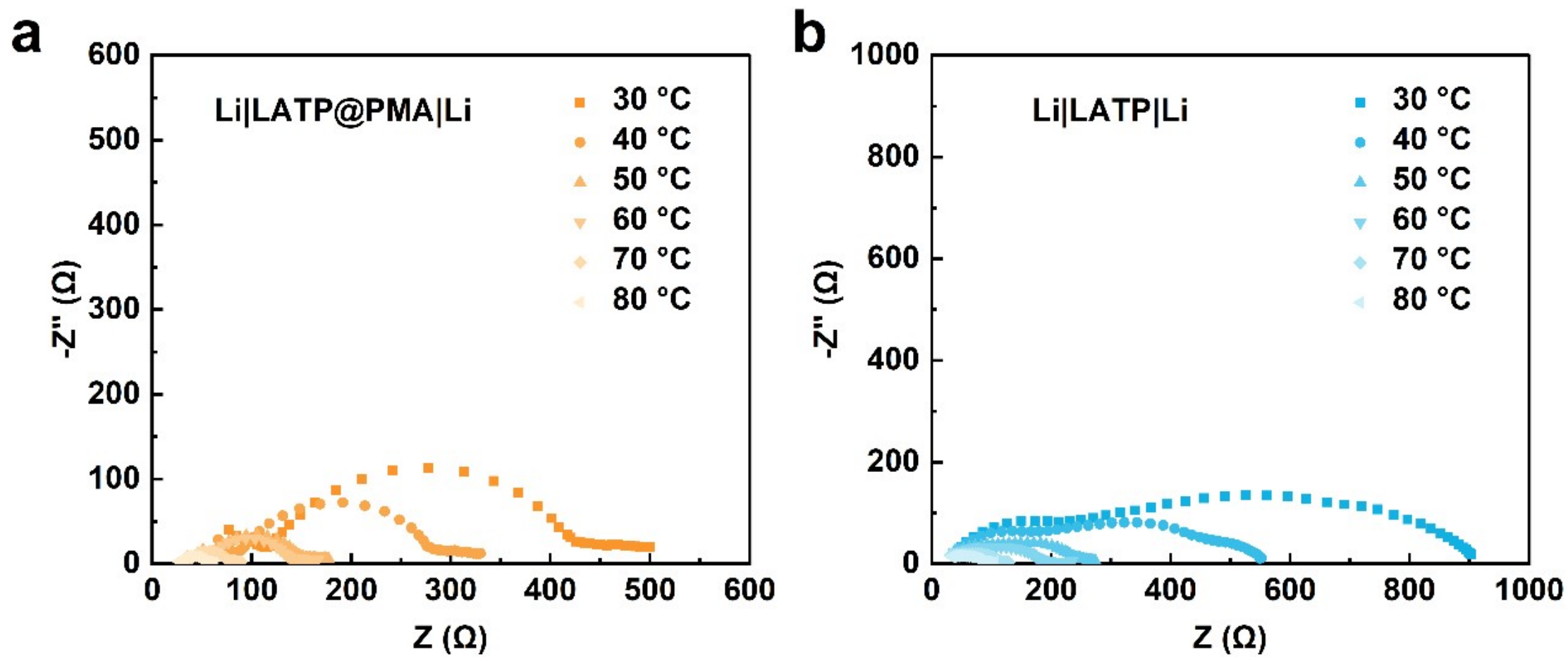

Fig. S12. Temperature-dependent electrochemical impedance spectra of the a) Li|LATP@PMA|Li and b) Li|LATP|Li symmetric cells measured from 30 to 80 °C.

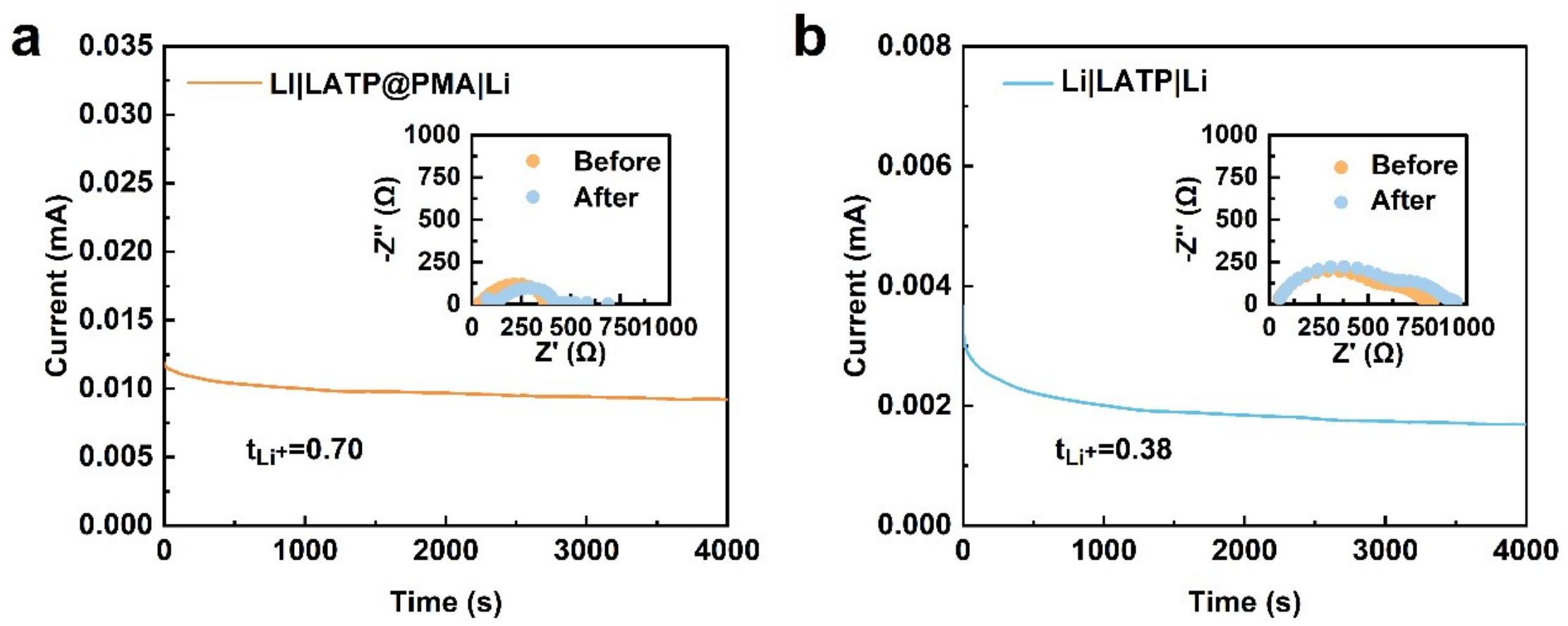

Fig. S13. Chronoamperometric polarization curves of the a) Li|LATP@PMA|Li and b) Li|LATP|Li symmetric cells. The insets show the corresponding impedance spectra measured before and after polarization.

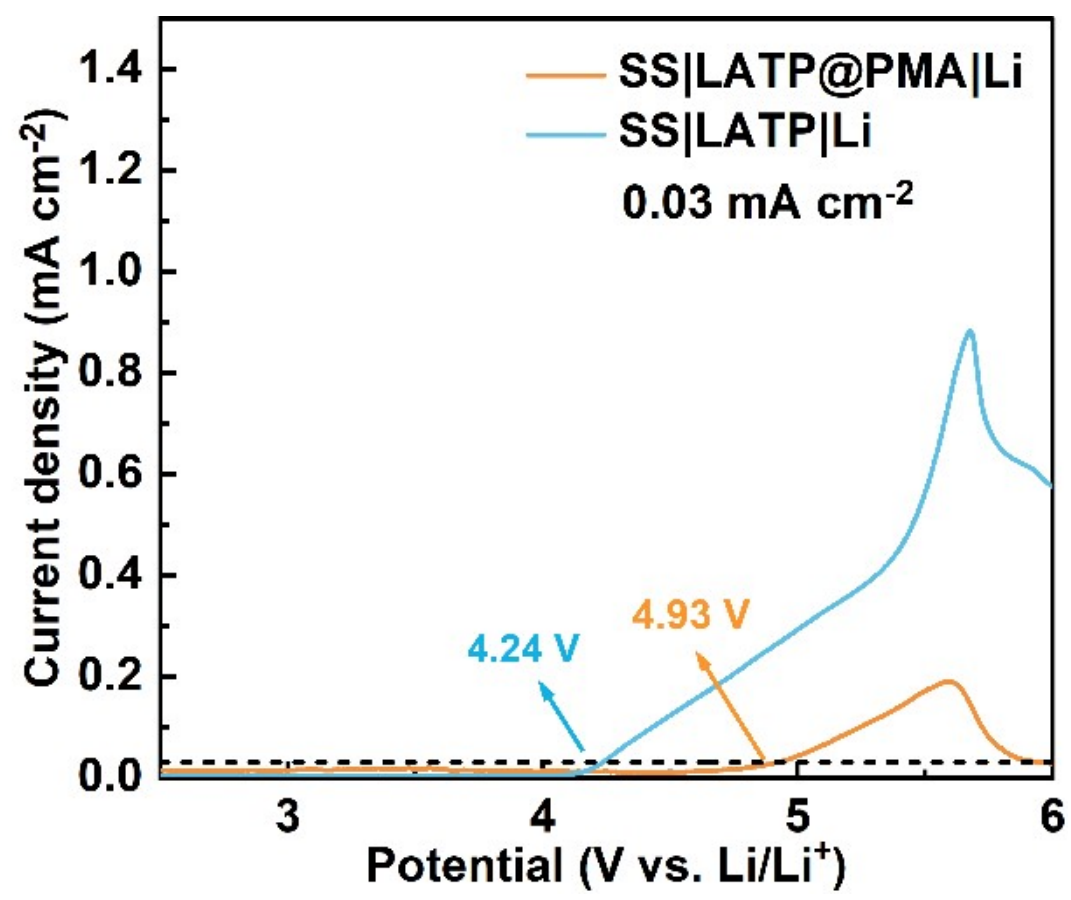

Fig. S14. Linear sweep voltammetry curves of SS|LATP@PMA|Li and SS|LATP|Li cells at 25 °C. The dashed line denotes the current-density criterion of 0.03 mA cm^{-2}.

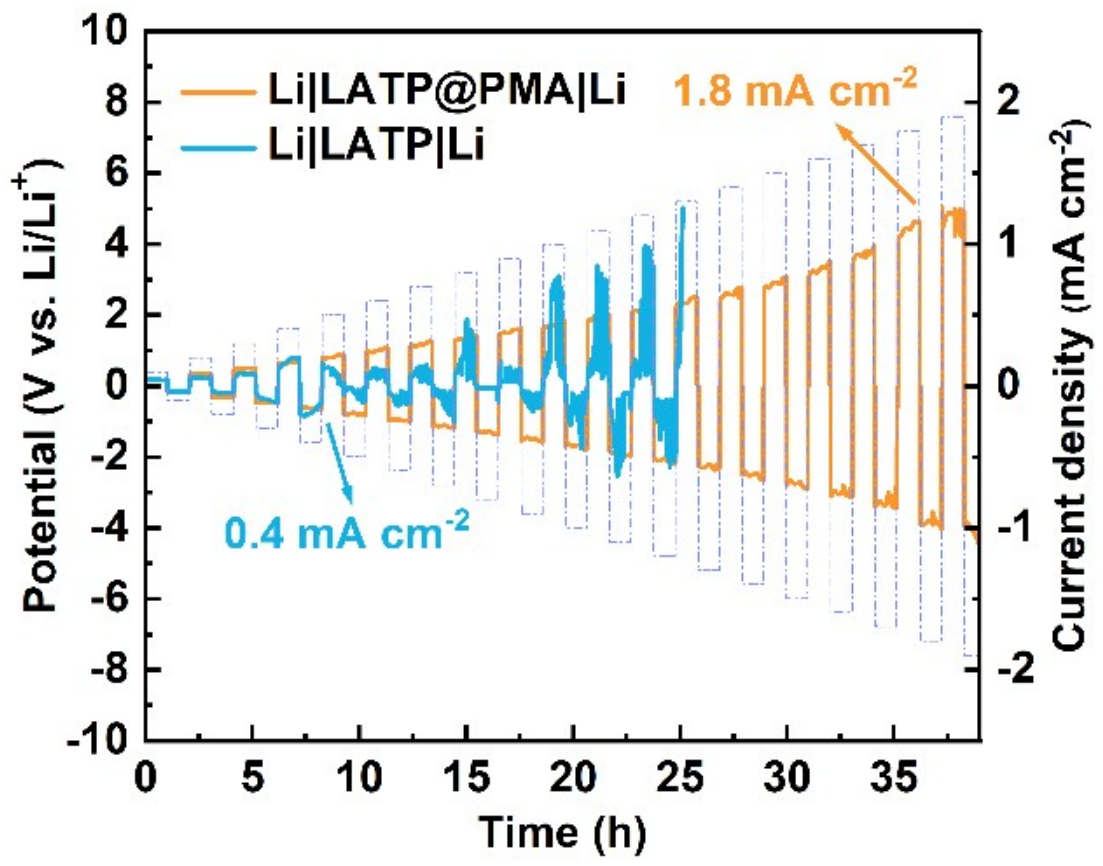

Fig. S15. Critical current density measurements of Li|LATP@PMA|Li and Li|LATP|Li symmetric cells at 25 °C.

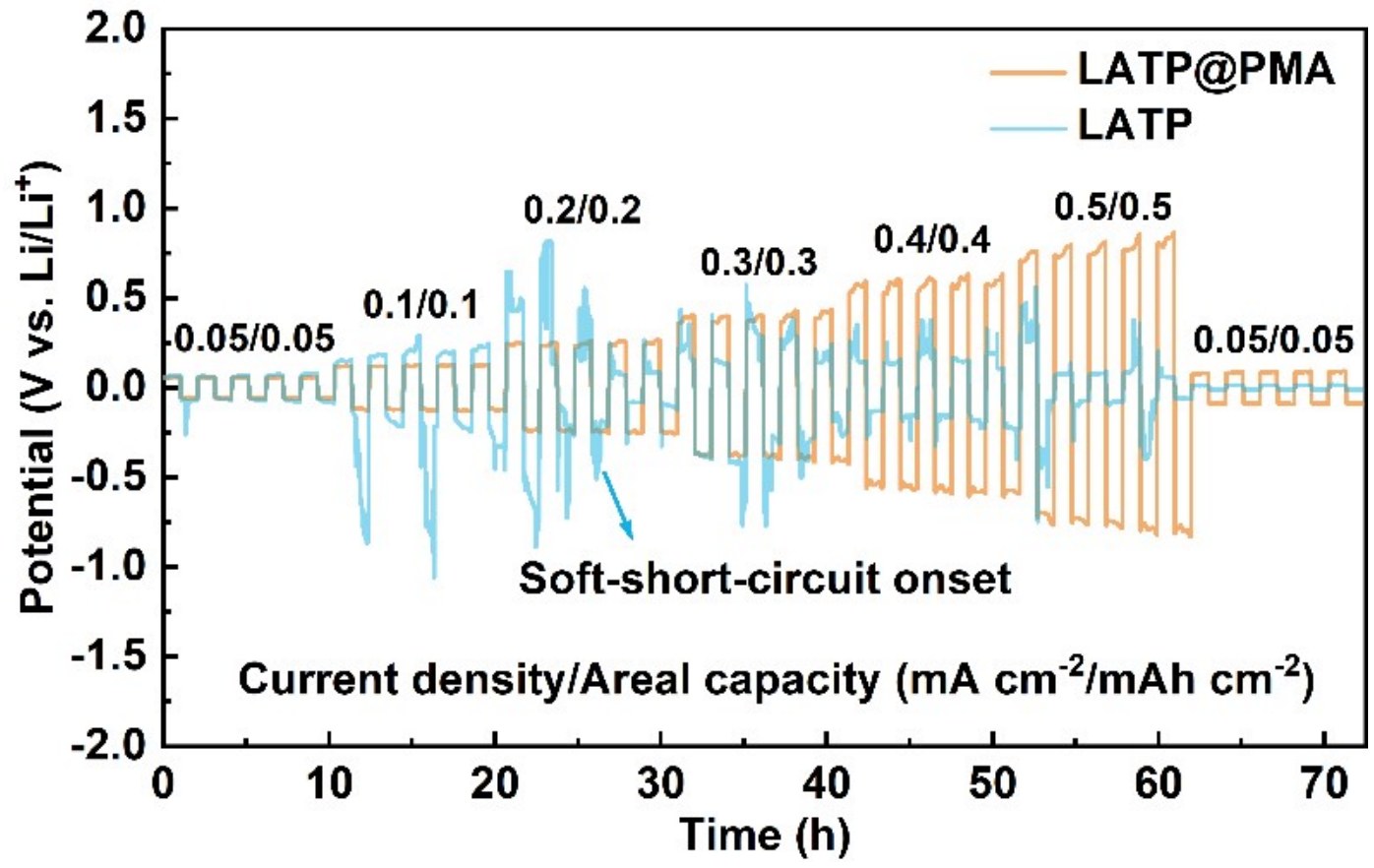

Fig. S16. Rate-dependent Li plating/stripping performance of Li|LATP@PMA|Li and Li|LATP|Li symmetric cells at 25 °C. The cells were sequentially cycled under current density/areal capacity conditions of 0.05/0.05, 0.1/0.1, 0.2/0.2, 0.3/0.3, 0.4/0.4, and 0.5/0.5, followed by a return to 0.05/0.05 (values in mA cm^{-2}/mAh cm^{-2}). Each Li plating or stripping half-cycle lasted for 1 h.

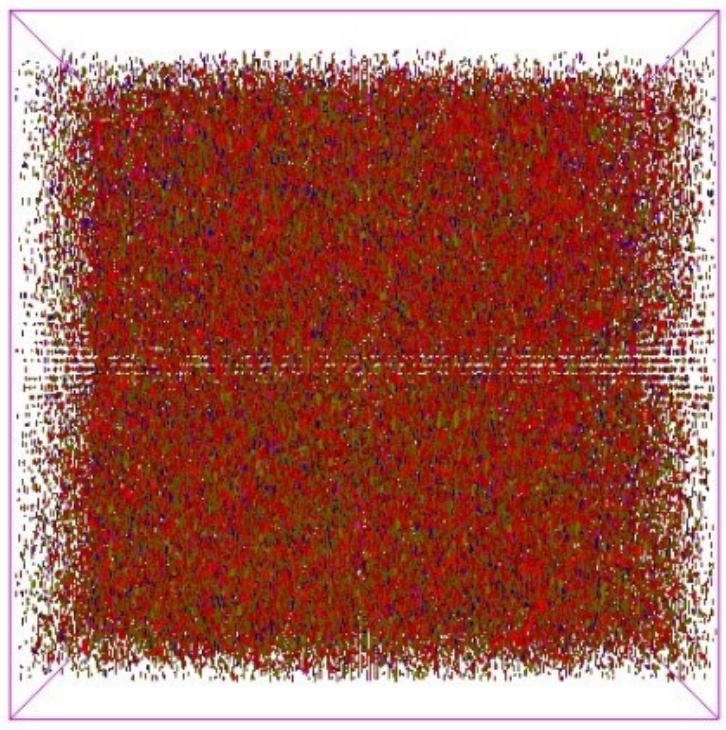

Fig. S17. Three-dimensional overlay of spatial distributions in LATP@PMA.

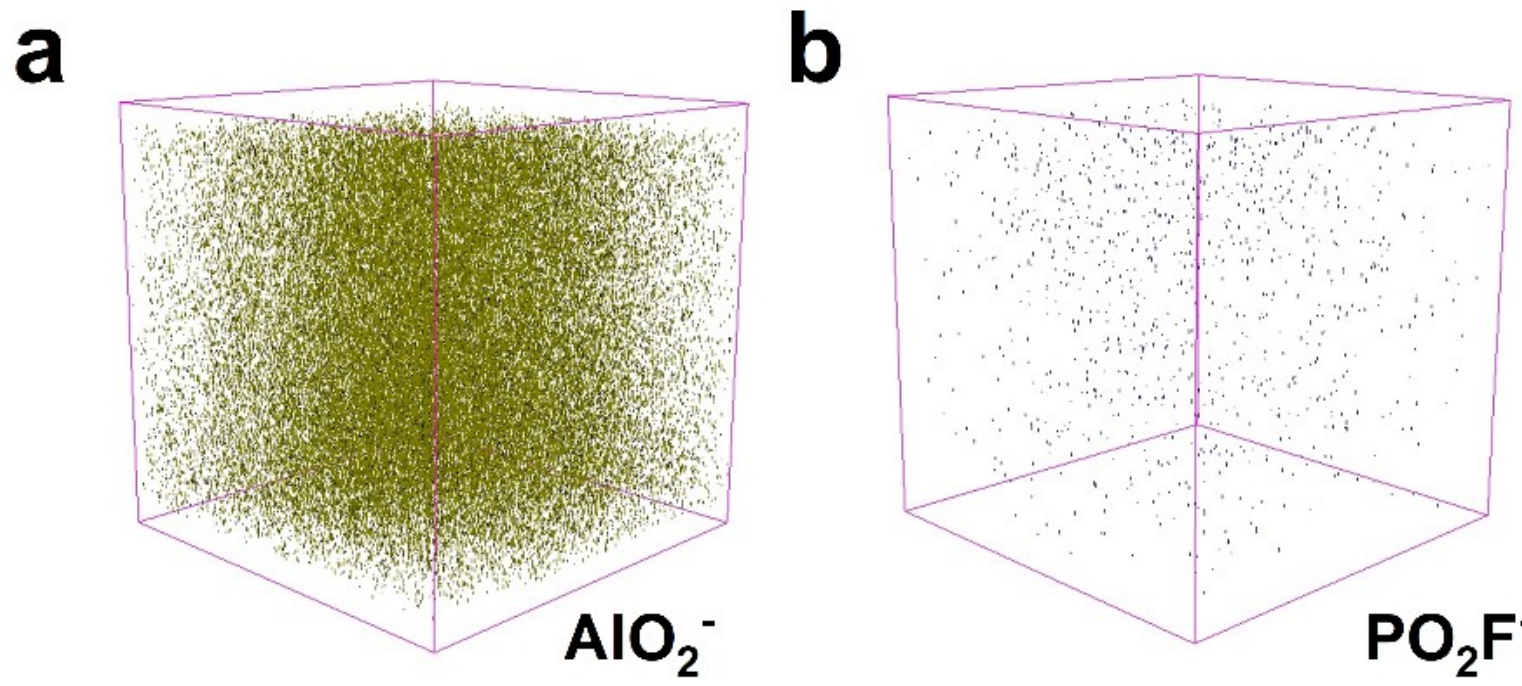

Fig. S18. Three-dimensional spatial distributions of a) AlO_2^- and b) PO_2F^- in the cycled interphase.

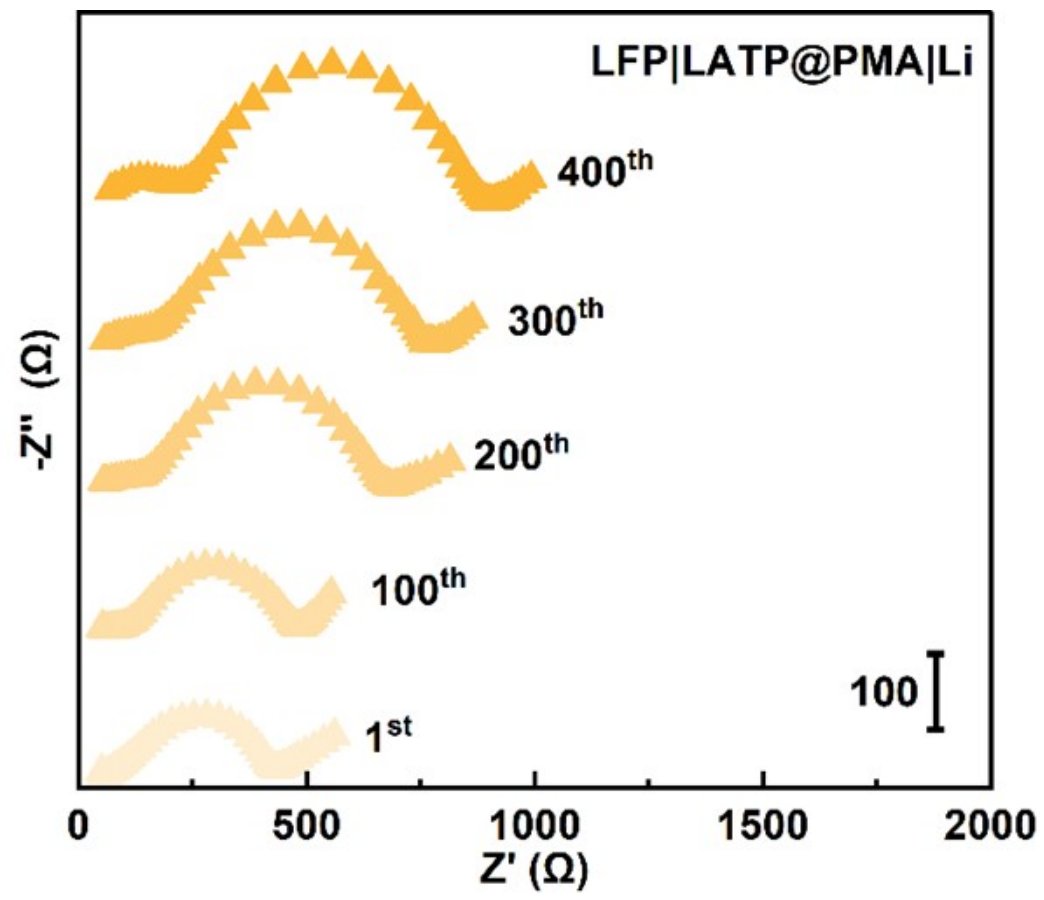

Fig. S19. Nyquist plots obtained from electrochemical impedance spectroscopy (EIS) for the LFP|LATP@PMA|Li cell at different cycle numbers.

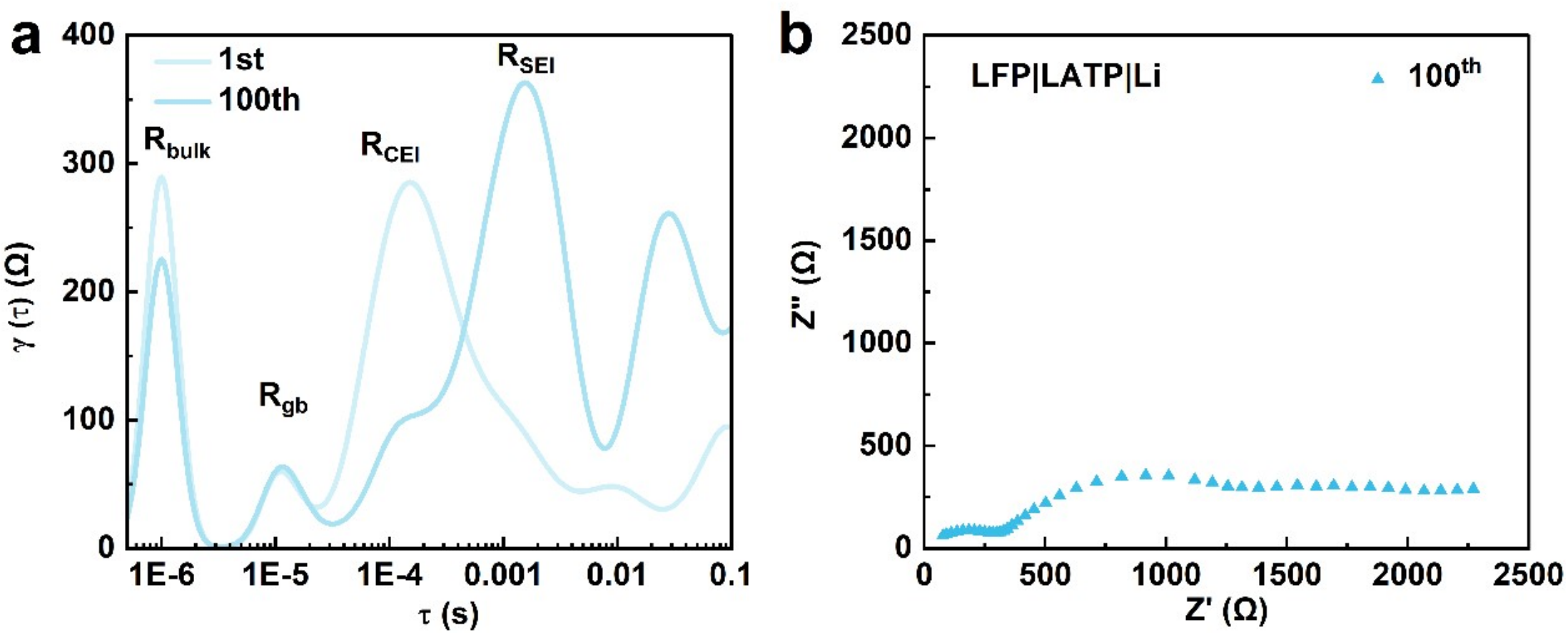

Fig. S20. a) DRT spectra of the LFP|LATP|Li full cells at different cycle numbers; b) Nyquist plots obtained from electrochemical impedance spectroscopy (EIS) for LFP|LATP|Li full cells at the 100th cycle.

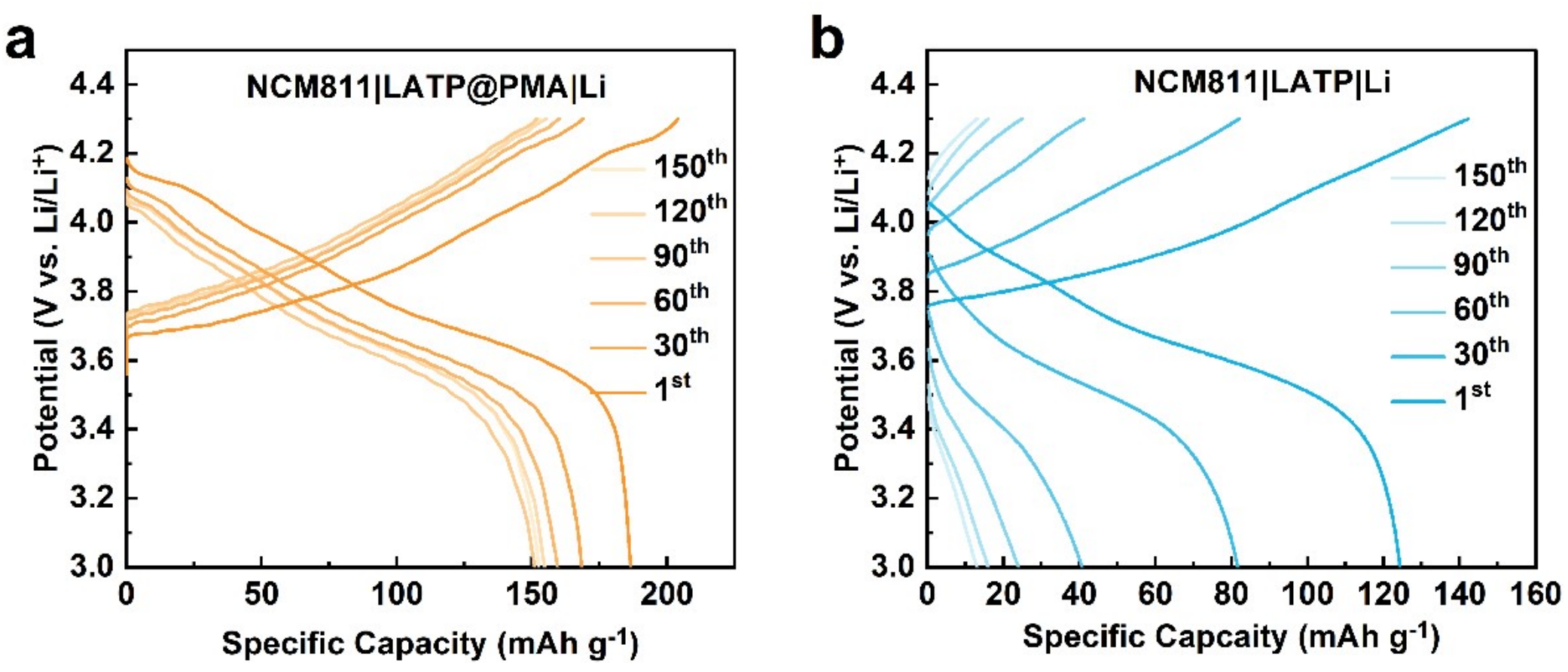

Fig. S21. Representative charge/discharge profiles at selected cycle numbers during the long-term cycling measurements shown in Fig. 7a: (a) NCM811|LATP@PMA|Li and (b) NCM811|LATP|Li cells.

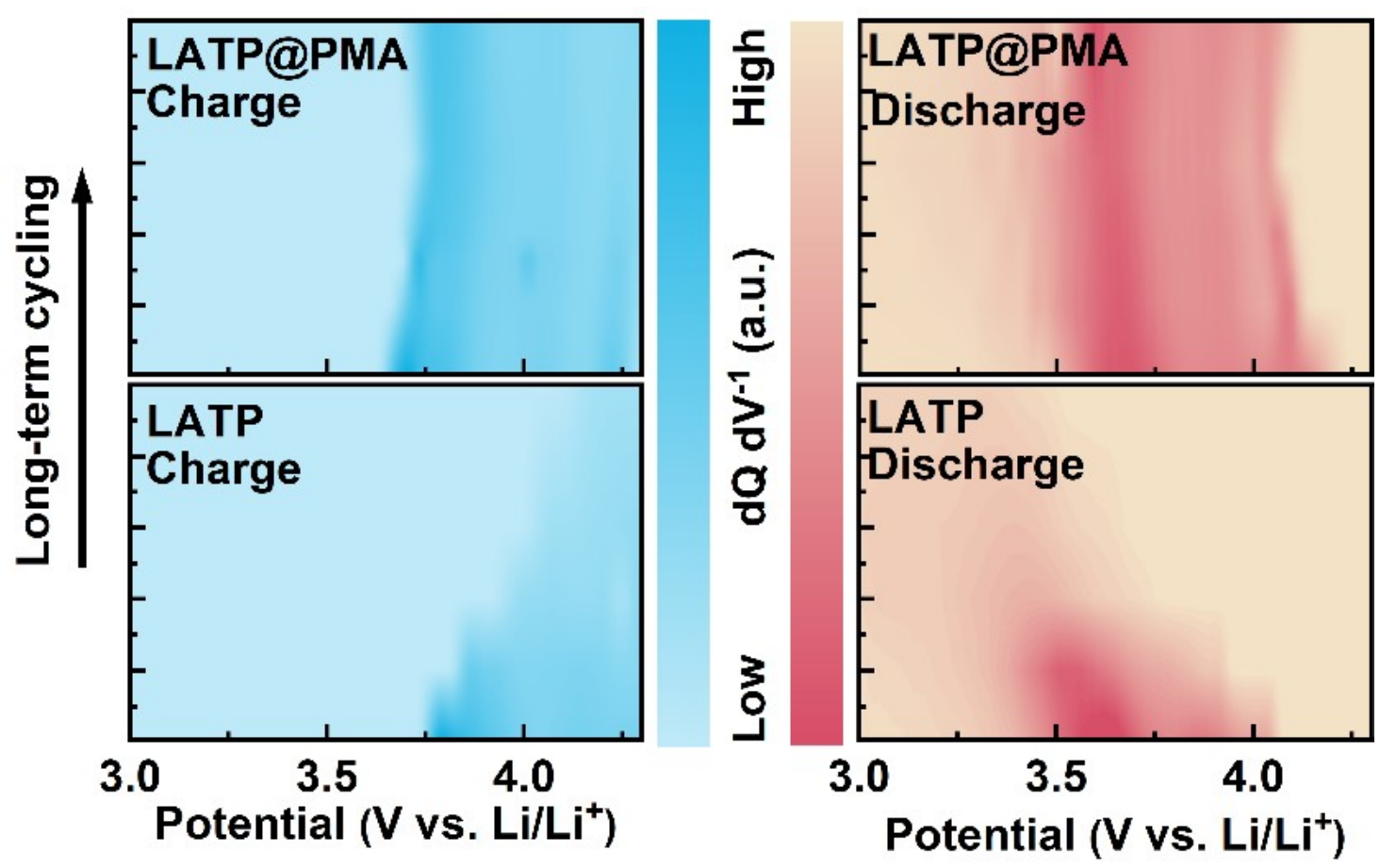

Fig. S22. Cycle-resolved dQ/dV contour maps of the charge and discharge processes for NCM811|LATP@PMA|Li and NCM811|LATP|Li cells during prolonged cycling.

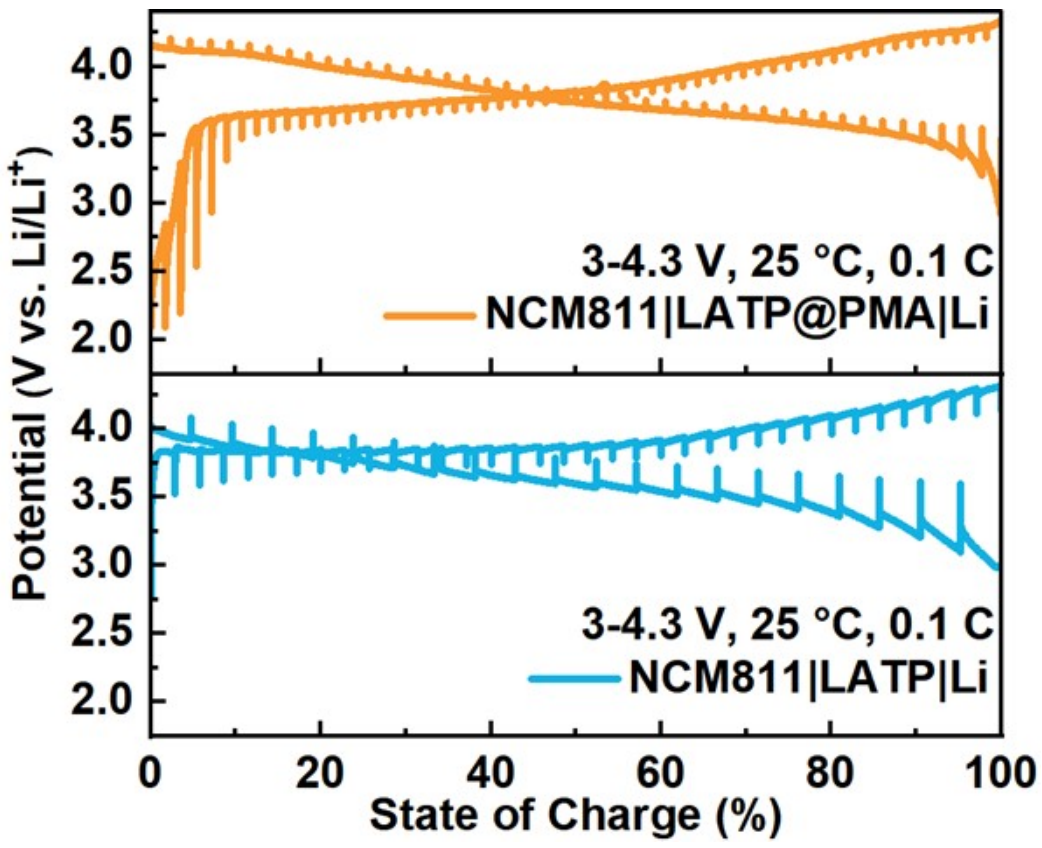

Fig. S23. Galvanostatic intermittent titration technique (GITT) curves of the NCM811|LATP@PMA|Li and NCM811|LATP|Li full cells.

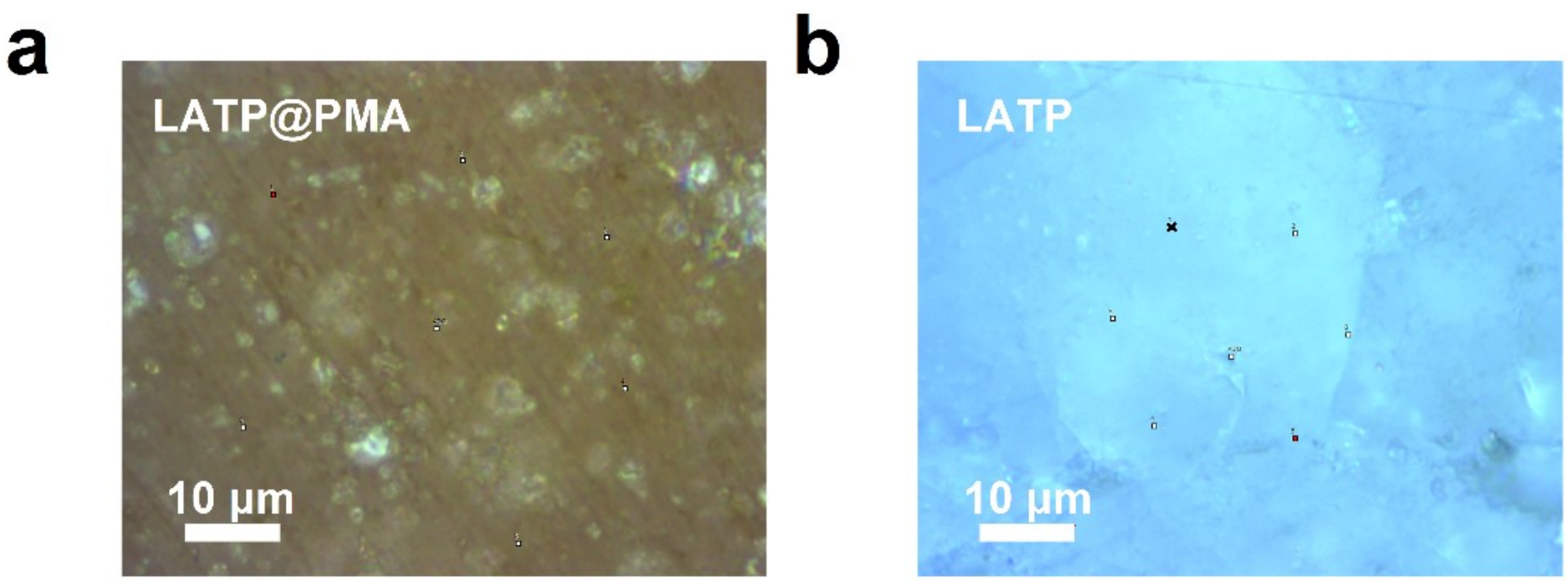

Fig. S24. Optical micrographs showing the spatially separated nanoindentation locations on a) LATP@PMA and b) bare LATP. Six locations were initially selected on each sample. All six measurements on LATP@PMA yielded valid results, whereas position 1 on bare LATP did not yield a valid Oliver–Pharr fitting result and was excluded from the statistical analysis.

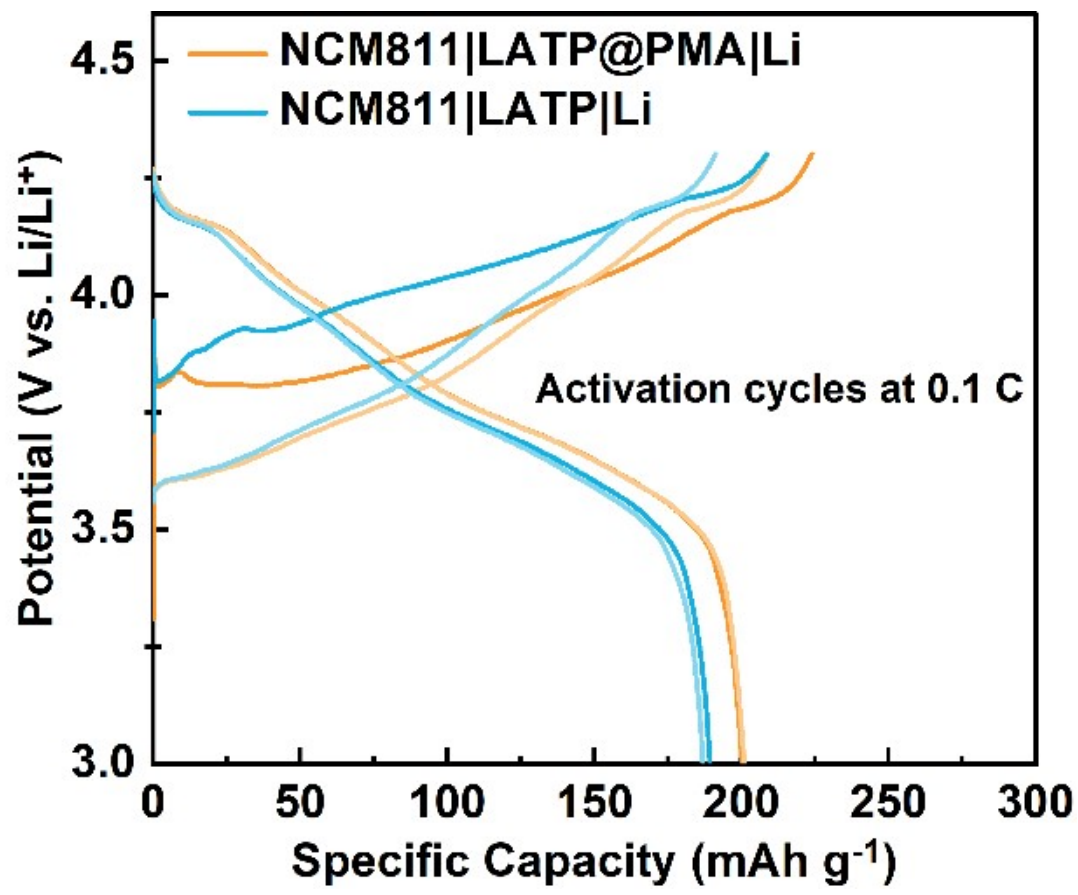

Fig. S25. Charge/discharge profiles of the NCM811|LATP@PMA|Li and NCM811|LATP|Li cells during the two-cycle protocol at 0.1 C before disassembly for post-cycling structural characterization

Table S1. Electrochemical performance comparison of full batteries based on LATP electrolyte interfacial modification.

**Cathode**	**Voltage range (V)**	**Rate (C)**	**Discharge specific capacity (mAh g^{-1})**	**Cycle number**	**Retention**	**Reference**
LFP	2.5-4.2	0.5	131.8	400	90.4%	$SnCl_4$[9]
NCM811	3-4.3	0.2	177.1	100	74.5%	
LFP	2.5-4	0.5	150.9	500	92%	BNRA[10]
LFP	2.5-3.8	0.3	139.2	500	96.6%	BN/PEO[11]
NMC	2.5-4.25	0.5	142	100	92.9%	
LFP	2.5-4	0.5	145	600	70%	BST[12]
LFP	2.5-3.7	0.5	121.3	500	88.9%	Al-SPE[13]
LFP	2.7-4	0.5	134.3	250	99.2%	PEO-LZP[14]
LCO	2.7-4.3	0.5	131.8	100	95.4%	$InCl_3$-LCO[15]
LFP	2.5-4.2	0.2	132	540	80.3%	CuF_2-LFP[16]
NCM811	2.8-4.3	0.5	135	100	77.7%	
LFP	2.8-4.2	0.3	140	300	93.4%	GPE-LFP[17]
NCM523	-4.2	0.2	153.7	120	85.7%	PEO-LiTFSI@BN[18]
LFP	2.2-4.2	0.1	157.6	200	90.2%	SnO_2[19]
NCM811	2.75-4.25	0.1	162	100	88.3%	
LFP	2.5-4.2	0.5	146.8	800	96.62%	This work
NCM811	3-4.3	0.5	186.5	150	82.14%	

References

1 W. C. Oliver and G. M. Pharr, *J. Mater. Res.*, 1992, **7**, 1564–1583.

2 T. H. Wan, M. Saccoccio, C. Chen and F. Ciucci, *Electrochimica Acta*, 2015, **184**, 483–499.

3 G. Kresse and J. Furthmüller, *Comput. Mater. Sci.*, 1996, **6**, 15–50.

4 G. Kresse and J. Furthmüller, *Phys Rev B*, 1996, **54**, 11169–11186.

5 J. P. Perdew, K. Burke and M. Ernzerhof, *Phys Rev Lett*, 1996, **77**, 3865–3868.

6 E. Torres and T. P. Kaloni, *Comput. Mater. Sci.*, 2020, **171**, 109237.

7 P. E. Blöchl, *Phys Rev B*, 1994, **50**, 17953–17979.

8 S. Grimme, J. Antony, S. Ehrlich and H. Krieg, *J. Chem. Phys.*, 2010, **132**, 154104.

9 D. Ding, H. Tao, X. Fan, X. Yang and L. Fan, *Adv. Funct. Mater.*, 2024, **34**, 2401457.

10 L. Zhu, Y. Wang, Y. Wu, W. Feng, Z. Liu, W. Tang, X. Wang and Y. Xia, *Adv. Funct. Mater.*, 2022, **32**, 2201136.

11 Q. Cheng, A. Li, N. Li, S. Li, A. Zangiabadi, T.-D. Li, W. Huang, A. C. Li, T. Jin, Q. Song, W. Xu, N. Ni, H. Zhai, M. Dontigny, K. Zaghib, X. Chuan, D. Su, K. Yan and Y. Yang, *Joule*, 2019, **3**, 1510–1522.

12 G. Li, X.-A. Li, K. Zhu, Y. Wang, M. Chen, J.-K. Kim and Z. Chen, *ACS Appl. Mater. Interfaces*, 2025, **17**, 66707–66715.

13 L. Luo, Z. Sun, H. Gao, C. Lan, X. Huang, X. Han, P. Su, Z. Zhang, C. Li, W. Huang, Q. Wei, Q. Zhang, M. Wang and S. Chen, *Adv. Energy Mater.*, 2023, **13**, 2203517.

14 F. Wang, B. Yang, C. Huang, S. Huang, M. Liu and H. Jin, *J. Mater. Chem. A*, 2026, **14**, 5273–5281.

15 W. Xiao, J. Li, C. Miao, Y. Xin, S. Nie, C. Liu and M. He, *J. Colloid Interface Sci.*, 2023, **652**, 1447–1455.

16 D. Ding, H. Ma, H. Tao, X. Yang and L.-Z. Fan, *Chem. Sci.*, 2024, **15**, 3730–3740.

17 W. Lei, C. Zhang, R. Qiao, M. Ravivarma, H. Chen, F. B. Ajdari, M. Salavati-Niasari and J. Song, *ACS Appl. Energy Mater.*, 2023, **6**, 4363–4371.

18 S. Han, H. Guo, A. Meng, L. Zhu, W. Tang and J. Chen, *Batter. Supercaps*, 2025,

8, e202500386.

19 L. Wang, L. Wang, Q. Shi, C. Zhong, D. Gong, X. Wang, C. Zhan and G. Liu, *J. Energy Chem.*, 2023, **80**, 89–98.
